# An Updated Overview of the Emerging Role of Patch and Film-Based Buccal Delivery Systems

**DOI:** 10.3390/pharmaceutics13081206

**Published:** 2021-08-05

**Authors:** Shery Jacob, Anroop B. Nair, Sai H. S. Boddu, Bapi Gorain, Nagaraja Sreeharsha, Jigar Shah

**Affiliations:** 1 Department of Pharmaceutical Sciences, College of Pharmacy, Gulf Medical University, Ajman 4184, United Arab Emirates; 2 Department of Pharmaceutical Sciences, College of Clinical Pharmacy, King Faisal University, Al-Ahsa 31982, Saudi Arabia; anair@kfu.edu.sa (A.B.N.); sharsha@kfu.edu.sa (N.S.); 3 Department of Pharmaceutical Sciences, College of Pharmacy and Health Sciences, Ajman University, Ajman 346, United Arab Emirates; s.boddu@ajman.ac.ae; 4 School of Pharmacy, Faculty of Health and Medical Sciences, Taylor’s University, Subang Jaya 47500, Selangor, Malaysia; bapi.gn@gmail.com; 5 Centre for Drug Delivery and Molecular Pharmacology, Faculty of Health and Medical Sciences, Taylor’s University, Subang Jaya 47500, Selangor, Malaysia; 6 Department of Pharmaceutics, Vidya Siri College of Pharmacy, Off Sarjapura Road, Bangalore 560035, India; 7 Department of Pharmaceutics, Institute of Pharmacy, Nirma University, Ahmedabad 382481, India; jigsh12@gmail.com

**Keywords:** buccal delivery, mucoadhesive polymers, penetration enhancers, buccal patch, buccal film, manufacturing, nanoparticles, evaluation, clinical trials

## Abstract

Buccal mucosal membrane offers an attractive drug-delivery route to enhance both systemic and local therapy. This review discusses the benefits and drawbacks of buccal drug delivery, anatomical and physiological aspects of oral mucosa, and various in vitro techniques frequently used for examining buccal drug-delivery systems. The role of mucoadhesive polymers, penetration enhancers, and enzyme inhibitors to circumvent the formulation challenges particularly due to salivary renovation cycle, masticatory effect, and limited absorption area are summarized. Biocompatible mucoadhesive films and patches are favored dosage forms for buccal administration because of flexibility, comfort, lightness, acceptability, capacity to withstand mechanical stress, and customized size. Preparation methods, scale-up process and manufacturing of buccal films are briefed. Ongoing and completed clinical trials of buccal film formulations designed for systemic delivery are tabulated. Polymeric or lipid nanocarriers incorporated in buccal film to resolve potential formulation and drug-delivery issues are reviewed. Vaccine-enabled buccal films have the potential ability to produce both antibodies mediated and cell mediated immunity. Advent of novel 3D printing technologies with built-in flexibility would allow multiple drug combinations as well as compartmentalization to separate incompatible drugs. Exploring new functional excipients with potential capacity for permeation enhancement of particularly large-molecular-weight hydrophilic drugs and unstable proteins, oligonucleotides are the need of the hour for rapid advancement in the exciting field of buccal drug delivery.

## 1. Introduction

The buccal region is an attractive site for target-specific delivery of the active(s) on the mucosa for local and/or systemic effect by absorbing through the mucosal membrane barrier covering the oral cavity. In comparison to oral drug delivery, the mucosal lining of the buccal region has a few unique advantages. It is highly vascularized and displays a decreased enzymatic activity, less sensitivity, ease of administration and expulsion of dosage form in the case of undesirable effects, avoiding acid hydrolysis of the stomach and bypassing hepatic first pass-effect. It enhances bioavailability of the drug hence requires a minimum dose and precipitates less dose related effects than other routes of administration. In addition, buccal administration exhibits better patient adherence in contrast to other non-oral drug-delivery routes. This route is excellent for potent drugs especially targeted for acute conditions with rapid clinical response due to direct access to the jugular vein and for extended therapeutic effect. Hydrophilic, acid and enzyme susceptible proteins and peptides that cannot be delivered via oral route because of poor absorption can be alternatively administered through the buccal route. The main limitations associated with buccal drug transport are the smaller surface area (~50 cm^2^) and dilution of drugs due to steady secretion of saliva (0.5–2 L/day) [1,2,3]. Accidental swallowing of saliva may affect the bioavailability of the drugs whereas inadvertent ingestion of this delivery system can induce choking particularly in pediatrics, geriatrics, and patients with dysphagia. The application of such a delivery system also restricts regular food intake and hence causes discomfort to the patients. The main formulation challenges faced by the scientists in designing buccal drug-delivery systems for systemic effect are due to limited absorption area, salivary restoration cycle, masticatory effects during eating and from the membrane barrier layers of the mucosa [4,5].

Many review articles have been published during the last few decades signifying the importance of human buccal mucosa as an attractive site for drug delivery [2,6,7,8]. The main objective of the current review is to highlight the recent advancement of nanocarrier-based buccal drug-delivery systems and various strategies to overcome the formulation and drug-delivery challenges involving buccal mucosa. The beneficial structural aspects of relatively immobile buccal tissue and less harsh oral environment recognize it as a potential and practicable site for the placement of mucoadhesive dosage forms. Nevertheless, a thorough understanding of anatomy and physiology of the oral cavity, permeability barriers, oral conditions and drug transport mechanisms involving buccal epithelia are imperative for the design, and development of buccal-based delivery systems.

## 2. Anatomical and Physiological Features of the Oral Cavity

**Figure 1:**
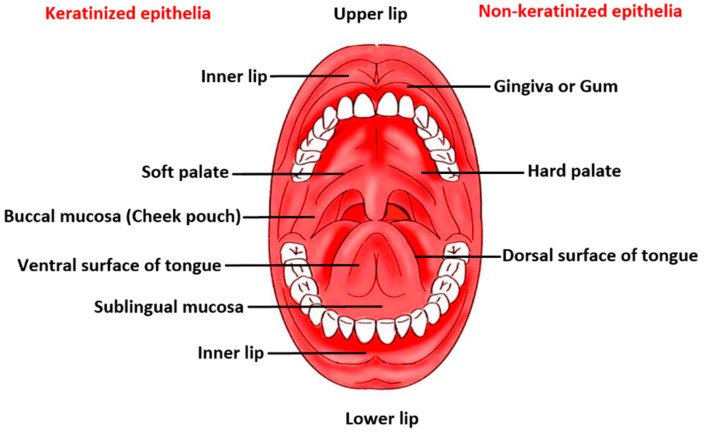
A schematic diagram depicting the key regions of the buccal area.

The anatomy and physiology of the oral mucosa covering a total area of 170 cm^2^ reveals three distinctive layers, namely epithelium, lamina propria, and submucosa. The protective buccal epithelial membrane is divided into flexible non-keratinized mucosal surface lining the soft palate, ventral surface of the tongue, sublingual mucosa, floor of the oral cavity, inner lips, and buccal pouch, and keratinized mucosa, which covers the hard palate, gingiva and dorsal surface of tongue in the oral cavity [9]. A schematic diagram displaying key regions of the buccal area is given in Figure 1. There are several text and reference books available that extensively reviews the main anatomical and physiological aspects of the oral cavity, teeth, tongue, salivary glands and orofacial muscles [10,11,12]. A summary of buccal mucosa and oral environment as a barrier for drug permeation/penetration are described henceforth.

### 2.1. Permeability

The epithelial membrane thickness is variable depending on the location for instance, the lining of the mouth and gingival surface is thicker (200–500 μm) compared to floor of the mouth (100–200 μm) [6]. The superficial epithelial cells (200 μm) consist of intracellular vesicles/organelles known as membrane coating granules or lamellar granules that generate specific types of lipids based on the location. Nonpolar (e.g., sphingomyelin, glucosylceramides, ceramides) and polar lipids (e.g., cholesterol esters, cholesterol, and glycosphingolipids) derived from membrane coating granules exist in keratinized and non-keratinized epithelium, respectively. These contents are discharged into the intracellular spaces of the upper epithelium, which significantly influences the permeability of substances [9,13,14,15]. Thus, the limit of permeation of actives can be estimated at the level where the membrane coating granules could be found bordering the superficial stratified epithelial cells. The tight junctions observed in intestinal and nasal mucosa are absent in buccal mucosa while gap junctions present are enriched with desmosomes and hemidesmosomes. It is, therefore, approximated that the transporting ability of the buccal mucosa is several fold (4–4000 X) greater than that of the skin [13]. The permeability of materials through thin, non-keratinized sublingual mucosa is more than thick non-keratinized buccal and thicker keratinized palatal mucosa [16]. Various permeation studies have demonstrated that the flattened outer epithelial exhibits the main barrier to mucosal permeation of toxins, drugs, antigens and enzymes compared to the cell layers of submucosal region [17,18].

### 2.2. Oral Environment

Mucus is synthesized and secreted from goblet cells containing mainly water insoluble glycosylated peptides called mucins, which covers the entire surface of the oral cavity. The oligosaccharide chains contribute negative charge to the mucins through carboxyl and sulfate residues and can form three-dimensional (3D) hydrogel building blocks. The thickness of the mucins ranges between 50–450 μm and form a strong cohesive structure that will bind to the apical surface of the oral epithelium [19,20]. The mucin presents an additional barrier, and it can either enhance or decrease drug absorption depending on the type of carrier and drugs [21]. For instance, charged molecules interact with mucin through electrostatic attraction, hydrogen bonding or hydrophobic interactions and therefore hinder their transport through buccal mucosa. The buccal transport mainly involves the mucosal lining covering the pouch or cheeks and the upper and lower lips and the corresponding thickness of the buccal epithelium varies between 500–600 μm [16,22]. Based on microfluidic design, it has been suggested that spatial charge distribution is a critical factor that impacts the transport of molecules through the mucosal pathway and the design of drug-delivery vehicles with tunable transport properties [23].

Saliva is a biological fluid secreted mainly by submandibular, the parotid and the sublingual glands. High turnover of saliva can dilute the concentration of drug present at the absorption site apart from decreasing the retention time of the drug in the buccal cavity, resulting in reduced buccal absorption. The pH of the saliva usually ranges from 6.0–7.5; however, it can be still lower (~5.5) in the case of oral ulcers, fungal and periodontal conditions [24]. The variation of pH and salivary constituents along with the flow rate of the saliva can influence the buccal absorption and subsequent clinical effect. Various in vitro studies can be carried out in simulated salivary fluid typically composed of potassium phosphate (1.6 g), sodium chloride (2.4 g) and calcium chloride (0.16 g) in a liter and the pH adjusted to 6.8 using sodium hydroxide [25].

## 3. Drug Transport Mechanisms

Drugs can cross buccal epithelium by transcellular (intracellular) and paracellular (intercellular) transport mechanisms [26,27]. The transcellular route mainly involves moving across the stratified epithelial membranes by low-molecular-weight lipophilic compounds with optimum lipophilicity (log P 1.6–3.3) and is a complex phenomenon [28]. The paracellular route permits small low-molecular-weight hydrophilic compounds to permeate through the extracellular amphiphilic lipid matrix via passive diffusion, which is a major barrier, particularly, for macromolecular hydrophilic compounds such as peptides. In addition, the proteolytic activity of the surface linked enzymes such as aminopeptidases can pose a major obstacle for the buccal delivery of peptide-based drugs [21]. The enzymes are typically located either on the surface of the mucosa and/or within the intracellular compartments such as aminopeptidase, carboxypeptidase and esterase that can provide an additional barrier to drugs that permeate through the buccal epithelium. Depending on the type of transport mechanism, a drug may or may not interact with all the available enzymes present in the oral cavity. Nevertheless, the enzymatic barrier present in the oral cavity is less severe compared to the gastrointestinal tract. Clinical effect is observed once the drug(s) diffuse across various biological membrane barriers to attain desired concentration at the target site. After buccal administration, drugs must either transport through the mucosal epithelial layers to reach systemic circulation or remain at the target site in the buccal region to elicit a pharmacological effect [29]. The salivary pH influences the extent of ionization and subsequently affects the rate and extent of buccal absorption. Unionized fraction of the drug can be increased using various strategies to improve the permeability of drugs that are extensively ionized at the pH of the saliva. Dilution of drugs in saliva as well as salivary gland dysfunction may change the pharmacokinetics of the drugs such as absorption, which may ultimately modify their therapeutic efficacy in the form of onset of action [30].

## 4. Design and Formulation of Buccal Drug-Delivery Systems

Traditional buccal dosage forms frequently fail to maintain desired drug concentration level either on the targeted mucosal site and/or in the systemic circulation. The key formulation challenges are salivary renovation cycle and mechanical stress due to masticatory effect during eating and drinking [8,31]. This can shift the drug aside from the site of absorption hence decreasing the contact time and change in distribution kinetics of the drug. To sustain the therapeutic effect, it is essential to extend the intimate association between active(s) and the membrane barrier of buccal tissue. To address these issues, buccal delivery system should be designed in such a manner to remain at the absorption site for desired duration of time, enhance the drug permeation across the mucosa to systemic circulation or into submucosal epithelial layers unaffected by the impact of salivary flow, pH, electrolytes, and mucosal enzymes [32]. The components in the buccal dosage forms are mainly classified as mucoadhesive polymers, penetration enhancers and enzyme inhibitors.

### 4.1. Mucoadhesive Polymers

Polymer hydration and swelling owing to diffusion of water and ensuing mucin dehydration are the main driving factor for mucoadhesion. Swelling should promote flexibility of the polymer chain and interpenetration between mucin chains thus reinforcing the mucoadhesive strength. The extent of spreadability and ability to form different types of intermolecular bonds at various hydration stages determines the characteristic of polymer to be used for buccal formulation. There exist many theories such as wetting, fracture, diffusion, electronic, adsorption and dehydration, which explain the mechanisms of adhesion between the polymer and mucin. According to wetting theory, work of adhesion and spreading coefficient can explain the mucoadhesion between the surfaces [33]. Quantitative measure of wetting of a material using contact angle goniometer can determine the mucoadhesion based on wetting theory. The force required to detach the mucosal membrane from the contact surface measures the mucoadhesive strength as proposed by fracture theory [34]. Texture analyzer and modified balance are typically used to determine the adhesive force between the two contact surfaces. Concentration gradient driven diffusion process involving mucoadhesive polymer and mucin determines the penetration rate and depth as interpreted by diffusion theory. The diffusion coefficient of the polymer relies on many factors such as molecular mass, viscosity, elasticity, crosslinking density, hydrogen bonding ability, charge, solubility, hydration, swelling and contact time [27,35,36]. Degree of hydration and swelling index measurement could evaluate diffusion-based interlocking between the polymeric platform to the mucus glycoprotein chain. According to electronic theory, attraction between oppositely charged surfaces causes electron transfer that leads to the creation of an electric double layer and subsequent mucoadhesion [37]. The adsorption theory explains mucoadhesion phenomenon as physicochemical interaction between the contacting surfaces because of strong primary bond or weak secondary intermolecular forces [38]. Dehydration theory defines the role of osmotic pressure in water movement causing dehydration of the mucosal layer and subsequent mucoadhesion with the polymer [39,40].

In recent years, various mucoadhesive polymers have been investigated for prolonging the retention time of dosage forms or actives at targeted sites of oral mucosa. The most frequently used polymers in buccal dosage forms include poly(acrylic acid) and its copolymers such as acrylic acid polyethylene glycol (PEG) monomethyl ether copolymer, polyvinyl alcohol (PVA), chitosan, sodium alginate, gelatin, carrageenan, hyaluronic acid [13,41,42,43,44], cellulose derivatives such as sodium carboxymethyl cellulose (NaCMC), hydroxypropyl cellulose (HPC), hydroxypropyl methylcellulose (HPMC), eudragit RS 100 [45]. Positively charged, biocompatible and biodegradable natural polymer, chitosan has been widely exploited as mucoadhesive polymer because of its electrostatic interaction with the negatively charged O-linked oligosaccharide chain of mucin [46].

Highly effective technique such as thiolation has been attempted to improve the mucoadhesive property of polymers since tethered thiols have the capacity to form disulfide bridge with the cysteine residues in mucin [47,48]. However, the in vivo mucosal retention of thiolated polymer is short lived because the disulfide bonds formed between thiol groups and mucin are reversible. Mucoadhesive property can be synergistically improved by functional group modification via chemical conjugation to existing mucoadhesive polymer. Catechol end-functionalization strategy to enhance mucoadhesion property of non-mucoadhesive polymer was reported [49].

In the case of mussel adhesion on polar contact surfaces, the mussel adhesive protein uses its hydrophilic amino acid side chains such as 3,4-dihydroxyphenylalanine (DOPA) to form strong hydrogen bonds. The unique chemical composition and properties have led to the development of synthetic analogues for potential use as mucoadhesive for drug-delivery systems. An improved mucoadhesion has been reported from the mixed hydrogels prepared from chitosan and catechol-containing compounds, namely DOPA (3,4-dihydroxy-L-phenylalanine), hydrocaffeic acid, and dopamine [50]. An investigation was carried out to evaluate the mucoadhesive properties between catechol-cross linked chitosan and the mucin. The residual quantity of catechol tethered chitosan that were analyzed using surface plasmon resonance spectroscopy revealed four-fold mucoadhesion augmentation and nearly 10 h in vivo retention compared to unmodified chitosan and chitosan with poly(acrylic acid) after oral administration. The evaluation clearly showed that inherent mucoadhesive characteristics of polymers can be augmented by means of conjugating with catechol groups [51]. In a recent study, a mussel-inspired mucoadhesive buccal film prepared from PVA-DOPA has been investigated for mucoadhesion to hydrated buccal tissue and effective buccal delivery of dexamethasone loaded in poly(lactic -co-glycolic acid) (PLGA) nanoparticles [52]. The presence of a vibration absorption peak (1734 cm^−^^1^) for the C=O bond confirmed by Fourier-transform infrared, ultraviolet-visible, and 1H-nuclear magnetic resonance spectra proved the successful synthesis of PVA-DOPA polymers. Ex vivo studies in rat models demonstrated that this film can achieve strong adhesion to wet buccal tissues up to 38.72 ± 10.94 kPa. It was further disclosed that polydopamine-coated PLGA nanoparticles pass over both mucus layers and epithelial cells swiftly and later release drugs for either local or systemic delivery.

### 4.2. Penetration Enhancers

These agents permeate into the skin and interact with various skin components such as intracellular keratin and intercellular desmosomes to increase drug flux by reversibly decreasing the stratified epithelial barrier resistance. Penetration enhancers increase drug transport by either directly interacting with keratin of the epithelial cells, disrupting the intercellular lipids, proteins and/or other components of the epithelium. It may enhance diffusion coefficient of the drug, increase thermodynamic activity of the drug in the vehicle, and/or increase the partitioning of the drug in the buccal epithelium [53]. The rearrangement of the lipid matrix of the buccal epithelium due to electrostatic interactions between cationic polymethacrylate and HPMC derivatives was found to promote the paracellular delivery of insulin nanoparticles [54].

The compounds that could mainly benefit from the inclusion of penetration enhancers are proteins, peptides and hydrophilic, low-molecular-weight actives [55]. The different types of absorption enhancers include surfactants, bile salts, fatty acids, complexing agents, polymers, cyclodextrins, and miscellaneous compounds such as azone analogues. Different types of penetration enhancers, transport mechanisms, and important disclosures are depicted in Table 1. Though, combination of penetration enhancers typically demonstrates an enhancement of absorption, continuous use of these agents may likely cause local inflammation or tissue injury [56]. The selection criteria for these agents are based on the physicochemical characteristics of the actives besides being nontoxic, physiologically compatible, non-irritant, pharmacologically inactive and organoleptically inert. Most of the penetration enhancers show concentration-dependent effects such as pyrrolidones, alcohols, alkanols, sulfoxides, glycols, azones and surfactants [57].

Chitosan is considered to be a potential penetration enhancer for the transmucosal absorption of hydrophilic macromolecular drugs. Chitosan has the proven capacity to enhance the paracellular transport of macromolecules in a protonated state at a pH of <6.5. Though transient widening of tight junctions present in intestinal and nasal mucosa might be probable transport mechanism for chitosan, however, absence of these junctions in buccal mucosal regions is still to be explained [68]. The main limitation preventing extensive use of chitosan is low solubility at physiological pH of buccal mucosa and compatibility issues with anionic drugs and excipients. Presently, many derivatives of chitosan have been explored to increase the solubility and permeability of chitosan at different pH values without any precipitation due to drug-polymer complexation [69]. An investigation on the assessment of chitosan derivative as penetration enhancers on porcine cheek showed that methyl-pyrrolidinone chitosan has the best mucoadhesive and penetration enhancement properties in buccal environments [70]. The study also revealed that permeation of acyclovir was reduced by partial depolymerization and ceased after partial reacetylation of chitosan.

GRAS approved surfactants and bile salts included as absorption enhancers in buccal mixed micelle spray dosage form (Oralin^®^; RapidMist^®^, Generex Biotech, Toronto, Canada) containing insulin have been approved in a few countries. Various in vitro and ex vivo techniques that exactly simulate the in vivo buccal conditions to establish and compare the penetration enhancement properties of diverse compounds are urgently needed.

### 4.3. Enzyme Inhibitors

Degradation due to various enzymes existing in the oral cavity can be noted as one of the main formulation challenges involving protein and peptide-based drugs for efficient buccal transport. The inclusion of enzymatic inhibitors is considered to be an efficient technique to overcome the enzymatic barrier and subsequently improves the buccal absorption of these macromolecules. Understanding molecular structure of macromolecules and its susceptibility towards the corresponding protease enzymes is crucial for the stability of the macromolecule in the buccal environment and the selection of suitable enzyme inhibitor(s) [71]. Enzyme inhibitors such as aprotinin, bestatin, puromycin and a few bile salts protect protein and peptide drugs from degradation by either changing the functional properties of the buccal enzymes, modifying their conformation and/or hindering drug-enzyme interaction [72,73,74]. Binding of complexing agents with free metal ions such as Zn^2+^ and Ca^2+^ results in formation of nonabsorbable complexes and therefore allows free drugs to permeate through the buccal mucosa. The chitosan-ethylenediaminetetraacetic acid (EDTA) complex has been developed, which has mucoadhesive as well as metal complexing characteristics [75]. The EDTA covalently bound to chitosan can complex with metal ions, which are vital for the enzymatic activity of proteases and thus minimize presystemic elimination of peptide-based drugs. Results indicated that the polymer conjugate is capable of binding 2.01 ± 0.12 mmol of zinc per gram of polymer at pH 6.5. Since zinc is an important cofactor for aminopeptidase N, enzyme activity could be totally inhibited using 1.0% chitosan-EDTA conjugate incorporating leucine enkephalin as a model drug. Buccal delivery of peptide drugs via mucosa can be promoted by the use of anionic and cationic thiolated polymers such as poly(acrylates) and chitosan. The derivatization of these polymers showed improved inhibitory properties against peptidases [76].

Polycarbophil- cysteine showed predominant inhibitory effect than its unmodified form on the activity of isolated aminopeptidase N and aminopeptidase S present on intact buccal mucosa [77]. The inhibitory effect of polymer conjugate is due to the binding of L-cysteine and Zn^2+^ ions present in the structure of carboxypeptidase and aminopeptidase. Use of thiolated polymers in place of enzyme inhibitors in drug-delivery systems can localize the inhibitory effect and therefore avoid increased enzymatic activity because of feedback regulation. The impact of various types of poly(carbophil), poly(acrylic acid) and chitosan-based thiomers on the permeation of model compounds across freshly excised different animal mucosae were compared in Ussing-type chambers. Significant permeation enhancement of hydrophilic model compound, rhodamine was noticed with chitosan-thiobutylamidine (0.5%) conjugate in combination with permeation mediator, glutathione (5%) compared to unmodified polymer [78]. Due to large molecular mass, thiolated polymer conjugates are not absorbed from the mucosal surface thus avoiding any systemic side effects. Therefore, combinations of thiomers with low molar mass permeation enhancer(s), an improved paracellular drug uptake for extended duration could be accomplished. The oral application of peptide hormones such as insulin and salmon calcitonin were demonstrated in in vivo studies using anionic thiomers, polycarbophil-cysteine and poly(acrylic acid)-cysteine (450 kDa) as drug carrier matrices [79,80]. Based on these results, thiomers are likely to be considered to be emerging novel multifunctional polymers for transmucosal peptide delivery.

## 5. Buccal Patch

Buccal patches have gained tremendous attention in drug delivery owing to superior patient acceptance mainly contributed by ease of application, thinness and elasticity that induces only negligible discomfort to the patient. Moreover, drug delivery via buccal patch offers a safe and convenient mode of drug administration, because drug absorption can be instantly aborted in the case of undesirable effects by discarding the formulation from the oral cavity. Buccal patches are non-dissolving, matrix modified release dosage form typically laminated and composed of nonporous backing layer and a drug-incorporated mucoadhesive layer, which bonds to the oral mucosa, gingiva or teeth. The drug is delivered in a unidirectional or bidirectional manner either into the submucosal layers, oral cavity or both [81]. Different types of release exhibited by the buccal patch and important highlights are characterized in Table 2. The contact angle measurement was suggested as a useful and rapid screening technique to identify potential mucoadhesive capacity of buccal formulation [82]. The buccal patch was fabricated from silica elastomer and various concentrations of carbopol 934P. The contact angle was measured with ophthalmic shadow scope and mucoadhesive strength was determined using the INSTRON. The method provides adequate information about the adhesive strength of the patch applied on a freshly excised rabbit buccal mucosa. The systemic delivery of thyrotropin-releasing hormone, RP-56142 (lauroyl derivative of a tripeptide), octreotide acetate, oxytocin, buserelin, calcitonin and leuenkephalin from buccal patches have been investigated [74].

Triamcinolone acetonide buccal patch prepared from different mucoadhesive polymers, namely carbopol, poloxamer and HPMC were evaluated to study its impact on duration of mucoadhesion, swelling ability, and solubility properties. Though having good mucoadhesive force, the main limitation of carbopol is high aqueous solubility to be used in matrix type of trans-buccal formulation. Poloxamer could decrease the aqueous solubility of carbopol without compromising the adhesive force owing to hydrogen bonding between these polymers [88]. Decreased swelling ratio and dissolution rate with increased adhesion time was noticed on the composite film constituted of carbopol and poloxamer. The release rate of triamcinolone from the patch was found to be highest with plasticizer, PEG 400 followed by triethyl citrate and castor oil. Frequently, composite mucoadhesive polymers are efficiently used to impart adequate mucoadhesive force, water uptake ability and residence time. Buccal patch fabricated from a combination of polymers such as chitosan and pluronic F127 at 2.9% *w*/*w* and 2.6% *w*/*w* were shown adequate mucoadhesive strength (3.58 ± 0.62 N), retention time (342.67 ± 17.21 min), water uptake at 1 h (24.53 ± 3.62%) and controlled release of metoprolol for 8 h [89].

Incorporation of acyclovir inclusion complex was found to significantly increase the percentage of drug release from the buccal patch. The molecular complex was prepared from hydroxypropyl beta-cyclodextrin and acyclovir in a fixed molar ratio (1:1). Though, the drug was more uniformly dispersed alone in the patch compared to patch containing inclusion complex, the advantage of enhanced permeation could enable it to develop as transmucosal buccal delivery of acyclovir [90]. The carvedilol buccal patch formulated with mucoadhesive HPMC E15 demonstrated high cumulative drug release (84.9 ± 0.09% release) and drug permeation (38.69 ± 6.61%) via porcine buccal mucosa in 4 h. Furthermore, bioavailability studies of carvedilol buccal patch in healthy pigs indicated two-fold improvement of buccal absorption compared to oral solution [91]. The potential role of buccal patches to deliver potent drugs targeted for acute therapy have been recognized but the capability to transport macromolecules to systemic circulation continues to challenge formulation scientists. Development of a chitosan buccal patch for insulin delivery employing ionic liquids -eutectic solvent as the permeation promoter has been disclosed [87]. Insulin was mixed with ionic liquid/eutectic solvent mixtures prepared using choline and geranic acid to create a viscoelastic gel and subsequently placed between bilayered chitosan. The safety and efficacy exhibited by such innovative technology built on buccal patch shows a promising future ahead for the challenging task of delivering hydrophilic macromolecules.

## 6. Buccal Film

Mucoadhesive films maintain strong adhesion with the mucosal membrane, spread over greater surface area, and thus cater accurate dosage, increase total drug absorption and therefore are well suited for local and systemic therapy. Biocompatible and biodegradable mucoadhesive films are most favored dosage form for buccal application due to their versatility, adaptability, physical flexibility, comfort, lightness, acceptability, ability to withstand mechanical stress, and adjustable size [92]. They have shown enhanced patient compliance compared to bioadhesive tablets due to ease of administration, enhanced bioadhesion until the duration of absorption, resulted in commercial approval by US FDA for buprenorphine, fentanyl, naloxone and lidocaine buccal films [93]. A few examples of successfully developed formulations of mucoadhesive buccal films by therapeutic category are tabulated (Table 3). Additional advantage is ease of scale-up because of adaptability and practicability of the film manufacturing process such as solvent casting, fused deposition modelling, semisolid extrusion, inkjet printing based on 3D printing technologies [94]. Such innovative techniques would likely decrease the preparation time as well as improve the mechanical characteristics of the films besides personalized manufacture of buccal film. Furthermore, customized multiple drug release profiles and individualized dosing is potentially feasible employing 3D inkjet printing methods.

Fast dissolving/disintegrating buccal film is ideal for pediatric, geriatric, psychiatric, bedridden or non-cooperative patients due to ease of administration, less risk of choking or suffocation, therefore guaranteeing patient safety [101]. The immediate drug release from the film will also allow rapid onset of action and decrease the time to reach steady state concentration. The main drawback of buccal films is the comparatively low dose of the active(s) that can be accommodated within a limited surface area; however multilayered printing using advanced techniques such as 3D printing may resolve this issue in near future [102,103]. Although research is continuing to progress in this area, 3D printing could address many formulations related issues by optimizing the critical formulation and printing variables to identify the printable design space. For instance, the dose limitation problem in buccal film could be solved by optimizing printing process factors such as droplet formation inkjet cartridges. Drug-delivery challenges due to incompatible ingredients could be minimized by fabricating films with compartmentalization using 3D printing. The use of inkjet printing and fused deposition modelling to produce drug-embedded buccal films has made significant progress in the last few decades [102,103]. Different strengths of warfarin (1.25 mg and 3 mg) orodispersible film for personalized dosing was printed using a modified thermal inkjet printer. The composition of the films was HPMC (20% *w*/*w*) and glycerol (3% *w*/*w*) [104]. Such individualized therapy films could be manufactured in a clinical setting that will maximize the therapeutic efficacy while minimizing adverse drug reactions. The practicability of the hot-melt ram-extrusion 3D printing for the preparation of maltodextrin orodispersible films was studied [105]. The optimum formulation and process variables to print a combination of maltodextrins/glycerin (80/20 *w*/*w*) are heating temperature: 85 °C; gauge size: 18 G; needle- foil distance: 0.6 mm; printing rate: 50 mm/s and angle filling: 120°. A proof-of-concept fused deposition modelling method for the fabrication of single and multilayered oral films has been investigated. The separation of taste masking and drug layers can potentially avoid many issues frequently faced in oral films [106]. Polymeric filaments constituting model drugs, paracetamol and ibuprofen were printed using polyethylene oxide and PVA at 90 °C and 130 °C, respectively. Single layered oral film had thicknesses of 197 ± 21 μm, and multilayered oral film had thicknesses starting from 298 ± 15 μm.

The mucoadhesive film constituted with Kollicoat^®^ IR and polyethylene oxide allowed higher drug loading of rizatriptan benzoate and propranolol hydrochloride [107]. It was found that inclusion of aloe vera gel powder as a natural permeation enhancer transported 73.22% of propranolol HCl and 96.11% of rizatriptan benzoate over 100 min through rat buccal mucosa without buccal mucosal damage. Due to the inclusion of generally regarded as safe nature of the excipient used to prepare buccal films, they have been suggested as an ideal drug-delivery system for the pediatric population [93].

Mucoadhesive matrix films comprising clotrimazole (10% *w*/*w*) designed for oral antifungal effects were prepared using hot-melt extrusion technique (HME) [108]. The main film formers used for building buccal film are HPMC and polyethylene oxide and the bioadhesive polymer included was polycarbophil. The films showed excellent content uniformity, drug content (93.3% ± 1.0), steady release and adequate bioadhesive strength (*p* < 0.05). Development and in vivo evaluation of domperidone controlled release buccal films by HME method using central composite design has been described [103]. The optimized film prepared from polyethylene oxide N750 and HPMC E5 LV demonstrated high mucoadhesive strength (3.86 kg/mm^2^) with maximum in vitro drug release (93.62 ± 2.84%) and drug permeation of 63.36 ± 2.12% at 6 h. Pharmacokinetic studies indicated significant enhancement of bioavailability from optimized buccal film (3.2-fold) compared to marketed tablets (*p* < 0.05). Improved dissolution and bioavailability with small molecular weight compounds have been demonstrated using HME technology [103,109]. Inclusion of plasticizer along with optimized processing conditions have exhibited potential ability to incorporate thermosensitive biologics in the extruded polymer matrices [110]. It is worthwhile to note that the permeability through buccal mucosa is many fold (4–4000) greater than that of the skin, but comparatively lower than the intestine. Insulin nanoparticles prepared from dimethyl ethyl chitosan impregnated in chitosan film for trans-buccal delivery have been described [111]. Ex vivo diffusion study using rabbit mucosa indicated that penetration of insulin was 17.1, 67.9 and 97.2% for chitosan, dimethyl ethyl chitosan and dimethyl ethyl chitosan nanoparticles, respectively. The investigation clearly reveals that thiolated-chitosan derivatives can potentially serve as a buccal permeation enhancer for many biologics.

**Figure 2:**
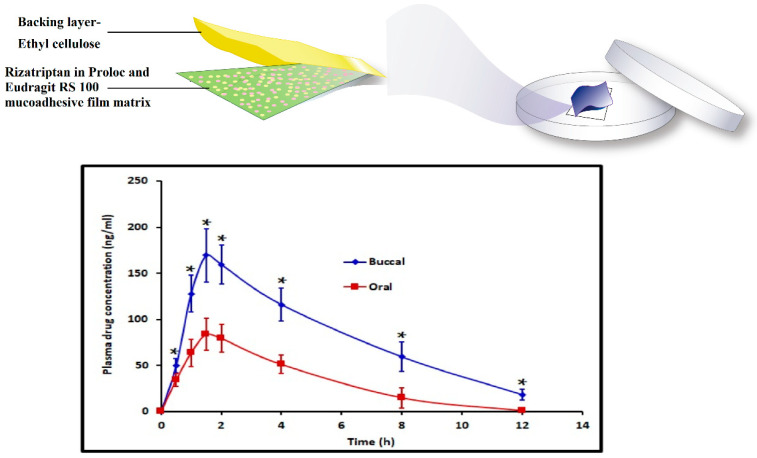
Development of rizatriptan loaded hydrogel-based mucoadhesive buccal film and enhanced buccal permeation displayed by the film compared to oral solution containing equivalent dose (adapted from [25], published by MDPI, 2021). *, Statistically different at *p* < 0.005.

In a recent investigation, we have successfully used bioadhesive, erodible polymer, Proloc 15^TM^ in combination with HPMC F4M and water insoluble polymer, eudragit RS 100 to develop buccal film for the efficient delivery of rizatriptan [25]. The optimized buccal film demonstrated significantly higher (*p* < 0.005) rizatriptan buccal flux (71.94 ± 8.26 ng/cm^2^/h) and AUC_0–12 h_ (994.86 ± 95.79 ng.h/mL) with a minimum lag time compared to oral solution with equivalent dose (Figure 2).

**Figure 3:**
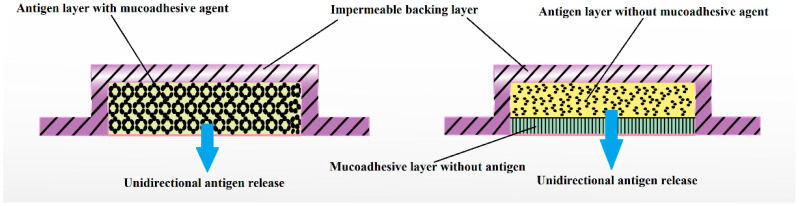
Possible designs of multilayered vaccine comprised buccal film with different functions for unidirectional release of antigen.

Buccal mucosa comprises dendritic cells and Langerhans cells, which make it an ideal site for the administration of vaccines. Films can deliver vaccines either in pure or particulate form to these cells for buccal delivery [112]. Vaccine formulations can be optimized in terms of antigen, size, surface potential, and specific receptor ligands particularly as particulate form would avoid probable degradation in the presence of saliva. It has been reported that the adsorption of antigens onto chitosan particles is a simple and effective loading process suitable for the product development of vaccines. Hydrophilic coating with sodium alginate to enhance the stability of nanoparticles and to hinder rapid release has been developed [113]. However, the main challenge during the development of such a process is to maintain the particle size in submicron size range and to facilitate these particles to be engulfed by microfold cells of the Peyer’s patches and transport to underlying mucosal lymphoid tissues. In vitro release studies in simulated intestinal fluid at 37 °C showed that the coating with sodium alginate was able to prevent immediate release of loaded ovalbumin. Such a strategy can be efficiently applied for the improvement of stability and trans-buccal delivery of various biologicals. The film dosage form of a vaccine can produce antibody mediated as well as cell mediated immunity. A multilayered buccal film accommodating vaccine can be suitably designed and developed for trans-buccal delivery. For example, a three-layered buccal film constitutes the inner mucoadhesive layer with permeation enhancing agent, middle vaccine layer and the outer slowly dissolving layer which allow the vaccine to move in unidirectional manner through the buccal mucosa (Figure 3).

Fast dissolving buccal film dosage forms could be developed as therapeutic vaccines for local or systemic administration. Many film dosage forms for oral administration have been developed by incorporating influenza vaccine, salmonella vaccine and 9-valent pneumococcal conjugate vaccine [114,115]. Due to the size, permeability and antigenicity issue, film dosage form with live vaccine may not be practicable. Live attenuated influenza virus (A/PR/8 strain, H1N1) was evaluated as a secure and efficient approach of activating defensive mucosal and systemic antibody responses against live influenza virus after administration through the sublingual route [116]. Encapsulating vaccines in polymeric drug-delivery carriers such as chitosan, PLGA and polylactic acid either as micro- or nanoparticles embedded in film also have the potential to enhance delivery, targeting, protection against degradation and controlled release of antigen at a specific site [117,118,119]. Targeted vaccine delivery using spray dried PEGylated nanohybrid system constituting lipid-PLGA nanoparticles containing antigen for enhanced cellular uptake and improved stability was reported [120].

Nanohybrid materials have the great potential for further development and use in the field of biomedicine. These unique classes of nanomaterials combine the beneficial properties of both organic and inorganic components in addition to specific advantages such as enhanced thermal and mechanical stability. Nanoparticles distributed in thin films have been investigated to circumvent the bioavailability issues of poor solubility and poor mucosal permeation associated with macromolecules such as insulin. Thiolated-chitosan nanoparticles enhance ex vivo diffusion of insulin by creating thiol disulfide bonds with mucin [111]. From above studies, the buccal area can be considered to be an attractive site for the delivery of vaccine because of its accessibility, avoidance of the first pass effect, and immunological advantages over other mucosal routes of administration. In the near future, attenuated and DNA vaccines could be potentially delivered through the buccal route. It is possible that an attenuated vaccine and DNA vaccine can be delivered via a film dosage form. In a recent investigation, oral dissolvable buccal film was fabricated from HPC, triacetin and pH enhancing agent (calcium carbonate) to encapsulate and stabilize live attenuated thermostable tetravalent rhesus-human reassortant rotavirus vaccine [121]. Preserved film vaccine demonstrated strong protection against virus shedding and diarrhea after being tested with a large dose of a virulent G1 HRV in gnotobiotic pigs compared to placebo and the reconstituted liquid oral RRV-TV vaccine. Multilayered buccal film with middle vaccine layer, outermost bioadhesive layer and innermost impermeable backing layer to allow unidirectional antigen release via subepidermal layers can be prepared by conventional solvent casting method. Antigens is generally incorporated in the polymeric solution with suitable viscosity and later cast onto a film application apparatus. Additional excipients should be added to withstand the temperature during the drying process and subsequent storage condition [122].

## 7. Functional Role of Nanoparticles in Buccal Drug-Delivery Systems

**Figure 4:**
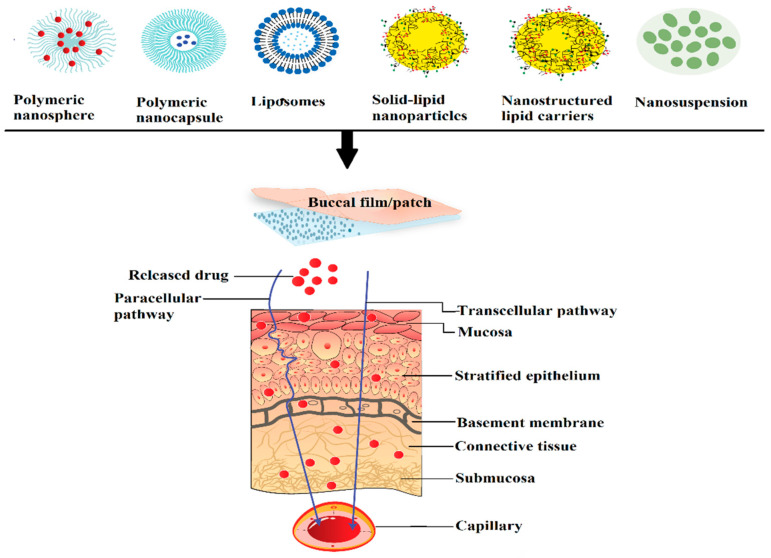
A schematic diagram showing the transport process of diverse nanocarriers through the buccal epithelium.

Nanocarriers are effective drug transport agents due to the possession of submicron particle size and unique physicochemical properties which would enable them to deliver to targeted tissues. They are versatile particulate, soluble or target-specific recognition moiety carrier systems capable of loading a diverse range of drugs prepared using different types of polymers and various manufacturing techniques. The use of nano-drug carriers can circumvent many limitations typically related to buccal drug delivery. Indeed, nanocarriers have many advantages such as enhanced diffusion coefficient of the drug through the mucosal epithelial layers, stability of the drug against degradation in buccal environment, prolonged contact time with the mucosa, extended buccal residence time by mucoadhesion, allowing for therapeutic concentration at the target site as well as decrease in the severity of the undesirable side effects [123]. Moreover, nanocarriers may minimize oral clearance, sustain or control the drug release, which decreases the frequency of administration and improves patient compliance. The composition of carrier as well as the entrapping agent determines the overall permeability, release rate, mucoadhesion to buccal epithelium and targeting ability. An ideal nanoparticulate carrier system must be capable of retaining close contact with the mucosa, thus allowing permeation enhancement of the actives without affecting the overall stability. Nanocarriers are generally formulated as aqueous dispersion or embedded within the matrix of the gel or film. Frequently used polymeric and lipid nanoparticles for buccal delivery and their characteristics are displayed in Table 4. An illustration exhibiting the transport of various types of nanoparticles via buccal mucosa is shown (Figure 4).

The aqueous salivary film covering the surface of buccal mucosa favors the absorption of hydrophilic actives fabricated as nanoparticles embedded on the hydrophilic polymer compared to lipophilic compounds [129]. Non-ionic or cationic nanoparticles have shown considerable mucoadhesion due to the interaction with negatively charged sialic acid, an essential component of the mucin. The close adhesion with the mucus layer causes prolonged residence time and resulting high concentration gradient of the drug at the application site. Despite this fact, the faster turnover times of the mucosal cells can limit the absorption of particularly lipophilic compounds. The physicochemical and viscoelastic properties of mucins besides being a variety of molecular interactions involving its complex and heterogeneous structure is of great significance in various mucin related diseases as well as in the design of efficient buccal drug-delivery systems [130]. Drug encapsulated nanoparticles move through epithelial barriers mainly by transcellular transporting mechanisms involving diffusion through the cells and paracellular mechanisms based on passive diffusion between cells.

It has been reported that the intact oral mucosa restrains the permeation of fluorescein isothiocyanate dextran (FD-20) and its permeation >0.6% per hour can be considered to be an indicator for barrier damage. The concentration-dependent penetration capability of bile salts on the delivery of polar large-molecular-weight compounds such as fluorescein isothiocyanate labelled dextran across porcine buccal mucosa was studied using confocal laser scanning microscopy [131]. The study indicated that the diffusion rate of permeation is influenced by the physicochemical properties of the drug and carrier besides the category and concentration of the penetration enhancers.

The insight into the fundamental understanding of the mechanism, by which protein stabilizes, will guide towards the formulation of protein and peptide-based pharmaceuticals [132]. Though, these biomolecules demonstrate high potency due to their large molecular size, short plasma half-life, the susceptibility to undergo degradation in both physical and biological environment, toxicity related to antigenicity, tendency for self-association, adsorption, and denaturation have restricted their ability to be developed as an oral dosage form. Since the biological membranes of the oral cavity show less proteolytic activity than the gastrointestinal tract, it can be considered to be a suitable route for delivery of peptides and proteins [21]. Insulin and enkephalin, also transported through the paracellular pathway without extensive metabolism because substrate enzyme are mainly present in cytosols. Nanoparticles have been investigated for the development of protein formulations due to the inherent ability to protect the therapeutic activity of macromolecules during the buccal uptake. Insulin loaded in chitosan nanoparticles was formulated based on an ionic gelation method using sodium tripolyphosphate as a crosslinking agent [133]. The prepared insulin loaded chitosan nanoparticles embedded film was light in weight (~23 mg) with minimum thickness (−0.32 mm), and exhibited sufficient mucoadhesive strength (2.3 ± 0.2 N). After buccal application, the prepared films were capable of significantly decreasing the blood glucose level in diabetic rats (*p* < 0.05).

### 7.1. Polymeric Nanoparticles

Hydrogels are flexible cross-linked 3D hydrophilic polymer structures capable of encapsulating small molecules and macromolecular drugs with controllable degradability. Self-assembled nanoparticles or nanogels, or hydrogel nanoparticles, have attained tremendous focus as emerging drug-delivery systems as it combines characteristic hydrogel properties with submicron particle size [134]. The polymeric nanoparticles can be efficiently used for the intracellular delivery of various therapeutic agents such as oligonucleotides, siRNA, DNA, and proteins [135,136]. The stability and wound-healing effect of mucoadhesive thermosensitive hydrogel comprised of trimethyl chitosan/β-glycerophosphate incorporating erythropoietin has been described [137]. The thermo-responsive property of hydrogel and structural stability of erythropoietin was successfully retained using the freeze-drying method. In a similar study, the best properties were demonstrated with the combination of trimethyl chitosan of 9.8% with a degree of substitution of 5% and glycerophosphate (20%). The erythropoietin loaded hydrogel reported in vitro/in vivo wound-healing properties was carried out with cattle buccal mucosa [138].

The inclusion of cellulosic or acrylic polymers generally results in rapid and prolonged bioadhesion even with high drug entrapment. Most commonly used hydrogel-based polymers for buccal delivery dosage forms are hydroxyethyl cellulose, carboxymethyl cellulose, HPMC, HPC, chitosan, polyacrylic resins and PVA, polyvinylpyrrolidone, Kollicoat, PROLOC™, maltodextrins, Lycoat NG 73, and pullulan [134,139,140,141]. Nanocarriers loaded into hydrogels are used for buccal delivery to improve the residence time, bioavailability as well as to protect the drug degradation. It was reported that salbutamol induced buccal epithelial changes were effectively counteracted by including a combination of bioadhesive poloxamer and xanthan gum in the buccal film formulation [142]. The cell viability enhancement evaluated using TR146 human buccal epithelial cell line indicates the applicability of these hydrogels for buccal delivery of diverse drugs.

**Figure 5:**
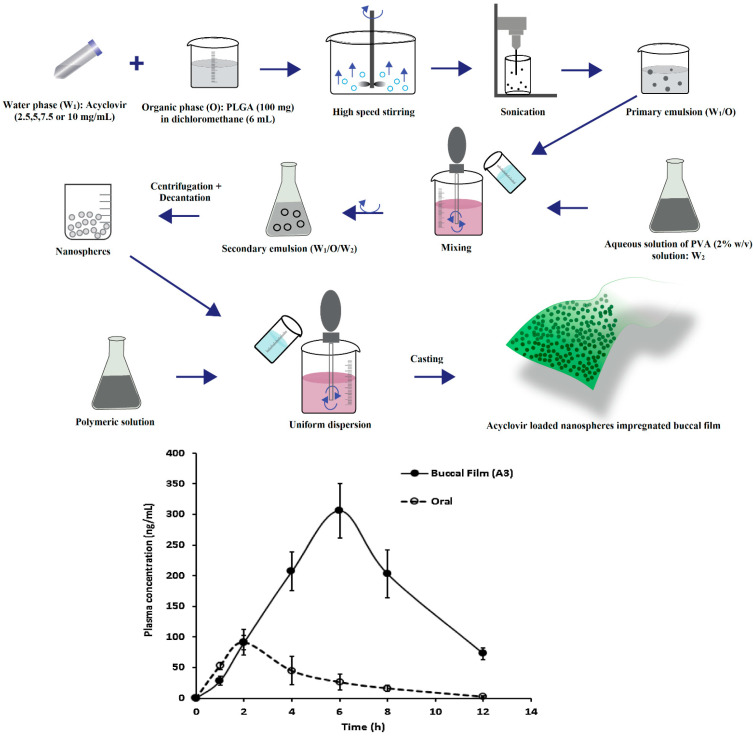
Development of acyclovir loaded nanospheres embedded buccal film and comparison of the plasma profiles of acyclovir following buccal application of buccal film and oral solution in rabbits (adapted with permission from [146], published by Elsevier, 2015).

Stimuli-responsive mucoadhesive hydrogels are an impressive choice for buccal therapy as they can modulate the drug release based on the response to external environmental changes and subsequent changes in structure, swelling capacity, diffusion or mechanical strength. The stability of susceptible drugs in the oral environment was improved by employing cationic hydrogel polymers such as chitosan [143]. The pH sensitive hydrogel with remarkable swelling characteristics was also observed with most frequently used synthetic polymers, acrylamide and methacrylic acid [144]. A major limitation identified with nanoparticles is uncontrollable and inconsistent initial burst effect, which induces huge loss of drug resulting in subtherapeutic drug concentration. This is mainly contributed by factors such as weakly bound actives, relocation towards the particle surface and complexity of the heterogeneous nano-matrix [145]. This behavior can be minimized to a certain extent by either particle-coating or matrix reformulation; however, it may change nanoparticle physical and chemical properties. The loading of carvedilol in bioadhesive gelatin nanoparticles and further incorporating in bioadhesive gel constituted of HPMC and NaCMC was found to decrease burst effect and significantly improved relative bioavailability through mucosal delivery compared to marketed product. The enhancement in the bioavailability could be possibly because of increase in drug solubility contributed by nanoparticles and avoidance of hepatic first pass effect. Ex vivo permeation investigations with excised rabbit mucosal membrane indicated that acyclovir nanospheres incorporated in matrix buccal film were effective in crossing the epithelial barrier [146]. It was demonstrated that *C*_max_, AUC, and t_max_ enhanced notably with the application of nanoparticles impregnated buccal film compared to buccal film loaded with drug. Acyclovir loaded PLGA nanospheres were prepared by double-emulsion solvent evaporation method and buccal film were fabricated by different concentrations of Eudragit RL 100, HPMC K15 and Carbopol 974P polymers. The acyclovir was rapidly absorbed after oral administration providing a *C*_max_ of 91.61 ± 42.88 ng/mL at 2 h while buccal film provides a maximum concentration of 3116.21 ± 246.37 ng/mL at 6 h. It was reported that buccal delivery markedly improves systemic availability of acyclovir (3116.2 ± 246.4 ng h/mL) as compared to oral solution as control (395.21 ± 64.20 ng h/mL) (*p* < 0.0001). It was suggested that delayed t_max_ (6 h) in comparison to 2 h after oral administration could possibly extend the duration of drug action and may decrease the number of drug dosing. A schematic diagram depicting the processing steps and in vivo pharmacokinetic evaluation in rabbits is shown in Figure 5.

The bioavailability enhancement observed with buccal film encapsulated with nanoparticles could be interpreted as mucosal permeation of these nanocarriers into the systemic circulation. Bioadhesive properties contributed by the film as well as nanoparticles can further extend the time of contact with the absorption site of the oral cavity. The release of nanoparticles from buccal film is essential before being delivered into and/or through buccal epithelium. It can be concluded from these studies that nanoparticles release from the film mainly relies on film matrix disintegration/erosion, followed by separation of nanocarriers. Furthermore, the drug release from the nanoparticles is dictated by solubility of drug, diffusion rate of drug through the soluble or insoluble carrier matrix.

### 7.2. Lipid Nanoparticles

Due to the diverse benefits of sustained and controlled drug release, high physical stability, low degradation of lipids, in vivo acceptability, and applicability to different administration routes makes lipid nanoparticles a very adaptive and effective carrier for various drug-delivery systems [147]. Lipid nanoparticles especially liposomes, solid-lipid nanoparticles (SLNs), nanostructured lipid carriers (NLCs), and nanoemulsions have the potential ability to entrap both lipophobic and lipophilic drugs, enhance the bioavailability of low aqueous soluble drugs, and protect them against untimely degradation. In addition, the lipids used to formulate the nanoparticles are safe, and thus demonstrate excellent tissue compatibility, and tolerability characteristics. Homogenization and sonication are the most frequently used techniques to prepare lipid nanoparticles [148].

#### 7.2.1. Liposomes

Due to their amphiphilic characteristics, liposomes have the capacity to entrap both lipophilic and hydrophilic actives. Liposomes loaded with pyridoxine and distributed in mucoadhesive film fabricated from HPMC and NaCMC were used for bioavailability enhancement of the drug [149]. Prolonged residence time of buccal film on the mucosal surface of the buccal epithelium was found to increase the penetration of the drug. The in vitro release studies of the buccal film embedded with liposomes showed extended release of vitamin B6 (72.7% after 105 min) in comparison to control film not loaded with liposomes (96.37% at 30 min). These studies also confirmed that the solvent casting method adopted for the development of the film did not modify the structure of the liposomes. The ex vivo permeability studies performed with vitamin B6 conjugated with liposome impregnated film using chicken pouch mucosa displayed slower flux (36.89%) related to vitamin B6 dispersed film and solution form of vitamin B6.

A three-layered buccal delivery system has been designed and developed with self-assembled liposome to improve the bioavailability of carvedilol [150]. The buccal film comprised of a liposome enabled electrospun layer, a bioadhesive layer and a backing layer. The ratio of both phospholipids to carvedilol and the molecular weight of polyvinylpyrrolidone had a significant influence on the drug encapsulation efficiency. The electrospun fiber constituting carvedilol showed excellent drug permeation compared to pure carvedilol. The in vivo pharmacokinetic study in rabbits showed 154% raise in the relative bioavailability compared to carvedilol suspension thus offered a novel platform for potential buccal delivery of drugs with high hepatic metabolism. In a similar manner, permeability studies disclosed that the liposomes considerably promoted the diffusion of silymarin through the buccal epithelial corresponding to silymarin solution [151]. A steady state drug permeation through the chicken cheek pouch was observed for 6 h. This study indicates that liposomes in buccal film can potentially enhance the drug permeation as well as extend the duration of action for a long period. Recently, an investigation was conducted to probe the biocompatibility, feasibility, and possibility of using insulin loaded liposomes comprising various bile salts to enhance the in vitro diffusion through buccal TR146 cell layers [152]. Flexible bilosomes encapsulated with insulin were prepared by a thin film hydration method using soy lecithin and bile salts. The formulated elastic bilosomes showed nano-sized particle size (~140–150 nm) and moderate encapsulation efficiency (66–78%). Bilosomes prepared using sodium deoxyglycocholate as edge activator reported marked permeation enhancement (5.24 folds) compared to other bile salts and insulin solution. The results obtained were further confirmed by fluorescence-activated cell sorting analysis and confocal laser scanning microscopy. From this study, it was concluded that cholate-based elastic bilosomes is a favorable means to increase the transport of insulin across buccal mucosa.

Quickly soluble film dosage forms can be considered to be ideal delivery vehicles for vaccines such as DNA-based liposome, bilosomes, and virus-like particles through the buccal or sublingual routes of administration. This would overcome the limitations associated with buccal mucosa due to rapid turnover of oral mucosal cells as well as the activity of enzymes, proteins and mucins that could curtail the achievement of these formulations. The crucial advantage of both buccal and sublingual mode of vaccine administration is the potential ability to produce both systemic and mucosal immunity. Both physical and chemical barriers hinder permeation of antigens through the epithelial layer to reach the antigen presenting immune cell.

#### 7.2.2. Solid-Lipid Nanoparticles

Recent investigation showed tremendous potential of nanoparticle-based buccal drug-delivery systems to furnish enhanced localization and drug targeting. The SLN is considered to be an efficient nano-drug-delivery carrier used for a broad range of drugs delivered through either oral or non-oral routes [147]. For successful buccal delivery, prolonged contact with SLN and buccal mucosa is important to minimize loss of drug due to saliva turnover, swallowing and chewing, tongue movements and phonation. Curcumin, a hydrophobic polyphenol has been shown to demonstrate antioxidant, anti-inflammatory, antimicrobial, anticarcinogenic, hepato- and nephro-protective, thrombosis suppressing, myocardial infarction protective, hypoglycemic, and antirheumatic activities [153]. To increase the residence time and mucoadhesion essential for the local therapy of precancerous lesions, curcumin-loaded SLN was dispersed in mucoadhesive poloxamer 407 gel. The SLNs were prepared by conventional melt dispersion technique followed by high-speed homogenization [154]. The results showed that the loaded gel with curcumin SLNs displayed excellent mucoadhesion and prolonged in vivo residence time (25 min). In vitro release testing by dialysis method revealed significant discrimination (*p* < 0.05) between percentage of drug released from curcumin-SLN gel (14.2%), conventional curcumin gel (27.7%) and curcumin-SLN dispersion (47.2%) after 5 h. Ex vivo permeation studies through chicken pouch mucosa reported enhanced permeation and localization of curcumin-SLN gel to reach basal epithelial cells, which is desirable to target curcumin in precancerous lesions. The amount of curcumin extracted from the bisected buccal mucosa was 21% after 3 h compared to curcumin solution (2% in 3 h) and curcumin- loaded SLN (18% in 3 h). Incorporation of curcumin in SLN in a gel matrix augmented drug penetration between mucosal layers due to intercellular lipid perturbation and alteration of tight junctions of epithelia contributed by poloxamer. It was concluded that curcumin encapsulated SLN in mucoadhesive gel matrix improved retention time, increased adhesion, permeation and localization of active through the basal epithelial cells of the buccal mucosa. Significant reduction of the lesion size and pain was observed in erythroplasia patients (*n* = 10) applied with curcumin-loaded SLN compared to patients treated with the curcumin gel without SLNs. Freeze-dried mucoadhesive sponges were also designed and developed to accommodate SLNs loaded with curcumin [155]. The curcumin-SLN was prepared from gelucire and poloxamer 407 and subsequently thickened with different mucoadhesive polymers. The data indicated that the curcumin-SLN loaded HPMC, and polycarbophil sponges demonstrated 4, and 15 h in vivo contact time, respectively, releasing high concentration of curcumin into saliva. Mucoadhesive sponge is an efficient carrier to deliver lipid nanoparticles while maintaining its structural integrity. The intermolecular attractive forces are mainly due to either stronger primary hydrogen bonds or secondary weaker dispersion forces, and the mucoadhesion was additionally increased because of the solid-state property of the dosage form. Lyophilized mucoadhesive chitosan sponges were also used for buccal transport of insulin and buspirone, respectively [156,157]. The oral absorption of poorly soluble drug, cucurbitacin B was significantly improved using lipid nanoparticles constituted of phospholipid-bile salts-mixed micelles [158]. The nanocarriers were later dispersed in fast dissolving oral film fabricated from pullulan and plasticizer, PEG 400. Results of optimized formulation showed a uniform size nano-micelles with an average diameter of 86.21 ± 6.11 nm and electrokinetic potential of −31.21 ± 1.17 mV. The pharmacokinetic study in Wistar rats demonstrated that lipid nanoparticles dispersed in oral films significantly improved in vivo absorption properties and subsequent oral bioavailability enhancement of cucurbitacin B (*p* < 0.05) compared to oral suspension. This study concluded that lipid nanoparticles enclosed in oral film could serve as a novel platform for the delivery of low aqueous soluble drugs via oral administration. The advantage of nano-enabled films for buccal delivery of didanosine has been reported [159]. Didanosine SLNs were formulated by means of hot homogenization process and later size reduction was done by ultrasonication before being embedded into multilayered polymeric films fabricated from glyceryl tripalmitate and poloxamer 188. The characterization of SLNs showed reduced particle size (201 nm), desirable polydispersity index (0.168) and moderate zeta potential (−18.8 mV). The nanoparticle loaded films released the drug rapidly as compared to conventional film (56% versus 26% at initial hour). Higher adhesive and mechanical strength was noticed with normal film compared to the nano-enabled film. SLNs did not change the permeation rate (71.63 ± 13.54 µg/cm^2^ h versus 74.39 ± 15.95 µg/cm^2^ h) thus proving the feasibility of transmucosal didanosine delivery using nano-enabled monolayer multipolymeric films. Buccal permeation study also suggested the local and systemic effect of fluconazole-loaded SLNs [160]. It would be more exciting to explore various types of lipid nanoparticles in different mucoadhesive polymers with respect to concentration, release kinetics, penetration ability and duration of action particularly in animal models differ in species.

#### 7.2.3. Nanostructured Lipid Carriers (NLCs)

In NLCs, lipid is present in both fatty solid and oily liquid state thus allowing more entrapment efficiency for certain drugs, minimum drug discharge during storage, preventing drug decomposition, slower drug release while demonstrating similar biological toxicity such as SLNs [161]. NLCs fabricated from spermaceti wax (solid-lipid) and soyabean oil (liquid lipid) comprising triamcinolone acetonide were used for buccal delivery applying the Box-Behnken statistical design. The drug loaded NLCs displayed particle size less than 200 nm, negative zeta potential (−5.91 to −20.83 mV) and percentage encapsulation efficiency higher than 80% for all prepared formulations. The data demonstrated that the ratio of solid and lipid had a critical effect on the release rate of the drug and inclusion of the surfactant (tween 80) was found to promote dispersity and solubility of the nonpolar drug in the simulated saliva. The permeation rate of drug-embedded NLCs was more than the drug in soyabean oil and the Nile red loaded NLCs could be visible at second and fourth hour at the peak penetration depths of 90 and 140 µm by confocal laser scanning microscopic technique [162]. Domperidone loaded NLCs were prepared to enhance permeability across buccal and sublingual epithelial barriers. NLCs were prepared with palmitic acid (solid-lipid) and oleic acid (liquid lipid) in the ratio 9:1 to dissolve the highest possible quantity of domperidone using high pressure homogenization techniques. Particle size of drug encapsulated NLCs was 283.97 ± 2.25 nm with a polydispersity index of 0.176 ± 0.015 and electrokinetic potential of −37.37 ± 0.31 indicated good physical stability. In vitro permeability experiments using TR 146 cell layers indicated that 11.48 ± 7.19% of domperidone in the cytoplasm and 17.99 ± 2.24% in the basolateral region from an applied amount of 750 μg/mL. It was hypothesized that after topical application, NLC results in the formation of occluded film preventing the transepidermal water loss along with promotion of hydration effects that leads to broadening of inter-corneocyte gaps. These cumulative effects allow the permeation of drugs into deeper layers of the skin besides transportation through the transappendageal pathway [163]. Inorganic nanoparticulate agents such as silica, clay and metals are typically formed as either nanoparticles, nanotubes, or nano-rods/nanowires [164]. Functionalization strategy adopted for these flexible nanocarriers carriers cater surface modification, drug targeting, and modified drug release. Though biocompatible and adaptable structures of silica-based nanoparticles are suitable for oral administration, inability to adhere to mucosa limits its clinical application. However, they are good candidates to be incorporated in matrix film composition for efficient buccal delivery. Functionalized silica–lipid hybrid microparticles loaded with cinnarizine after oral delivery was found to improve the bioavailability by avoiding any recrystallization after dissolution and improved drug partitioning by creating a hydrophobic microenvironment [165]. Such strategy could also probably provide bioavailability improvement of slightly soluble drugs incorporated in buccal film.

### 7.3. Nanosuspensions

Nanosuspensions are preferred, when an active pharmaceutical entity has major limitations such as inadequacy to form salt, large molecular mass and dose, high lipophilicity and melting point that curb them in developing effective dosage forms. Increased drug loading with minimum dose volume, less usage of excipients and harmful toxic non-aqueous solvents, retaining in amorphous state, increased stability, sustained release, minimum first pass metabolism and increased efficacy through tissue targeting are other key advantages of drug-based nanoparticles [166]. Nanocrystals in buccal films would favor ease of administration, enhanced dose accuracy, and excellent and consistent performance. Nanosuspension incorporated in mucoadhesive buccal film is suggested as a state-of-the-art technology for delivery of drugs associated with high hepatic first pass effect and low aqueous solubility. Carvedilol nanosuspension incorporated in three-layered mucoadhesive buccal film, i.e., outer mucoadhesive, middle nanosuspension and an innermost backing membrane has been developed [167]. Nanosuspension exhibited a negative zeta potential (−17.21 mV) with mean particle size of 495 nm and a polydispersity index of 0.203. Nanosuspension was later added to hydrogel layer prepared from HPMC and carbopol 934P using PEG 400 as plasticizer before inserting between mucoadhesive and backing layers. In vivo studies carried out in rabbits displayed significant enhancement (916%) in bioavailability compared with commercial tablet dosage forms. The *C*_max_ (7.3-fold) and t_max_ (4 h) of the prepared buccal film was higher than marketed formulation mainly contributed by enormous surface area of nano-sized drug and bypassing hepatic first pass elimination.

Inclusion of nanosuspension directly in film may offer additional benefits such as reduced cost of therapy, preventing premature or exaggerated release of drug in the body, reducing plasma level fluctuation and interpatient variability. Unlike other methodologies, nanocrystals in nanosuspension could accommodate potentially all hydrophobic drugs to allow sustained release [166]. Recently, development and in vitro evaluation of the oral mucoadhesive films containing clotrimazole nanosuspension for oral candidiasis treatment was described [168]. Bottom-up technique was employed for the preparation of surfactant (benzyl succinyl chitosan) stabilized clotrimazole nanosuspension and later included in catechol-functionalized hyaluronic acid/PVA mucoadhesive film. The slow release of clotrimazole from the nanosuspension loaded film was noticed, and the complete release was attained at 6 h. Furthermore, films were nontoxic to the normal cells and indicated significant antifungal efficacy in comparison to clotrimazole suspension.

## 8. In Vitro Evaluation Techniques

The routine tests used to evaluate buccal film are thickness, weight variation, film endurance, flexibility, degree of water uptake and swelling, surface morphology, moisture content etc. (Table 5). In addition to their mucoadhesive characteristics, the structural integrity of the film is prerequisite for their performance after buccal application. Various tests are conducted to evaluate the mechanical properties of the film such as tensile strength, puncture strength, elongation at break, elastic modulus, porosity, and folding endurance typically based on the ASTM D882-01 method. Furthermore, the developed films are examined by scanning electron microscopy, X-ray diffraction, differential scanning calorimeter, Fourier-transform infrared spectroscopy etc. Puncture testing can provide data on how well the film can withstand the puncture propagation against the compression force until it fractures. Folding endurance measures the flexibility of the film and resistance to wear, which is critical during the manufacturing process and patient handling. Though difficult to isolate non-keratinized mucosa from the buccal tissue, the mucosal lining of the rabbit closely mimics human buccal membrane and has been extensively used in various ex vivo and in vivo research studies. More information regarding the in vitro evaluation of buccal dosage forms are reviewed elsewhere [139,169] The dose conversion between human and animal can be carried out using allometric equations [170]. In vivo absorption after nanoparticle transport after mucus barrier permeation or paracellular transport is more adequately predicted by an ex vivo mucosal permeation model than in vitro permeability tests using artificial membranes. The histological evaluation additionally offers biochemical, anatomical, and structural features that resemble closely to its in vivo counterpart [171]. In vivo drug absorption from nanoparticles investigated in rats, rabbits or humans are estimated by pharmacokinetic parameters viz the AUC, t_max_, *C*_max_. The nanoparticle tracking and biodistribution in tissues is mainly observed by tagging the particles with fluorescent dyes. An important point to note is that plasma drug concentration does not differentiate between buccal from intestinal absorption.

## 9. Preparation Methods, Scale-Up Process and Manufacturing Considerations

**Figure 6:**
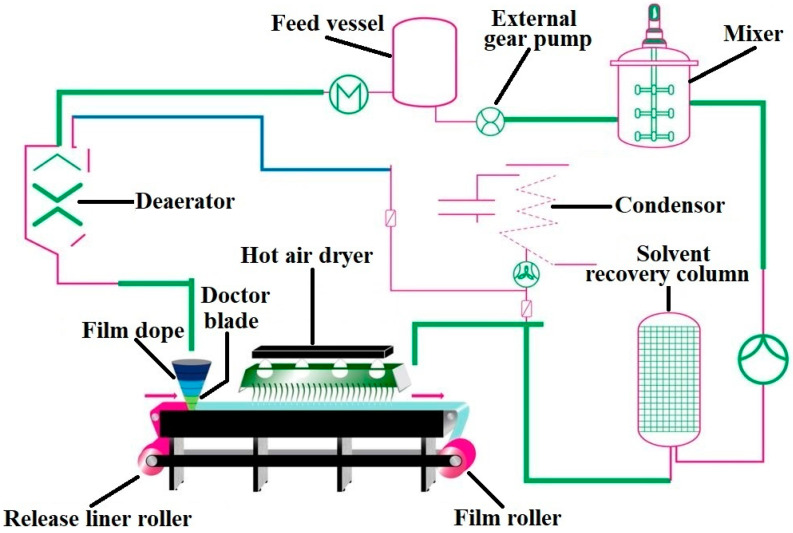
Flow chart illustrating the various processes typically involved in buccal film manufacturing based on solvent casting technique.

Presently the most extensively investigated technique for the preparation of buccal film is solvent casting technique [186] compared to hot-melt extrusion [187] and emerging 3D inkjet printing [188] method. Similar to manufacture of oral films, continuous fabrication of buccal film is efficiently adaptable for mechanization. Drug product critical quality attributes based on the quality target profile attributes can be used as a guideline for the film formulation and process development [189]. The main component of film is polymer (s), and its selection depends on the acceptable strength and stability of the film in addition to properties such as mucoadhesiveness, flexibility, moisture content, disintegration time and dissolution rate. Nontoxic non-aqueous class 3 solvents such as acetone, ethyl alcohol, isopropyl alcohol and non- toxic aqueous solvent, water is generally used to either dissolve or disperse the drug uniformly within the polymer film matrices [190,191]. Solvents are typically chosen based on the solubility of polymer as well as physicochemical characteristics of the active pharmaceutical ingredient. The type and concentration of plasticizer is important to enhance the elasticity of the film besides playing a key role in the dissolution rate of the film and process scale-up. Inactive ingredients such as surfactants are used as wetting, distributing and solubility enhancing agents, lipids as stabilizer for hydrophobic drugs, penetration enhancers for enhanced permeation, impermeable polymer as backing layer, organoleptic agents for better patient compliance. Flow chart illustrating the various processes involved in buccal film manufacturing based on solvent casting technique is presented in Figure 6. Solvent casting manufacturing method starts with accurately dispensing the drug, generally regarded as safe excipients, and solvents added in a proper sequence into a thermostatically controlled mixer and then mixed using an appropriate high shear or low shear mixer to ensure homogeneity. Nanoparticles should not be incorporated using a high shear mixer since it may disrupt the carrier, therefore releasing the encapsulated drug [192]. To ensure homogeneity of the mixture, samples should be taken from different locations of the mixer and measuring viscosity and drug content. In process microbial testing shall be carried out to check the possible bioburden in the slurry stored at appropriate environmental conditions. The slurry is then allowed to pass through a hot air oven set at appropriate temperature and applied to a carefully chosen liner by means of knife-over-roll coater at a measured pin gauge. Factors that influence the cast film formation are evaporation rate of the solvent, air flow velocity of hot air, location of the heat source, dimension of the pin gauge and speed of the belt [193]. The last step in the buccal film manufacturing process is cutting the master roll into single dose units, which are subsequently packed into individual pouches or sachets by packaging machines. Primary packaging material can be metalized polyester, which protects the dosage form from heat, light and humidity. The pouch material can be child resistant while the closure system is designed for both tamper resistant and user friendly. The dose of the drug within the buccal film is directly related to weight and therefore it is crucial to determine the weight of the individual film unit that is packaged. A key benefit with this dosage form is the simplicity by which multiple dose units can be generated by easily modifying the size of the film.

## 10. Clinical Translation of Buccal Administered Molecules

Buccal films were successfully developed for local effects and small drug molecules for systemic effects. However, slight progress with buccal delivery has been accomplished so far for macromolecules and biologics. The clinical development of buccal insulin delivery formulation, PharmaFilm^®^ embedded with gold glycan-coated nanoparticles bound recombinant human insulin was unsuccessful because of low buccal insulin bioavailability [194]. Ongoing and completed clinical trials of various actives targeted for trans-buccal delivery are summarized in Table 6. Though, various formulation challenges involving biologics continues to remain difficult as ever, the feasibility of buccal route for delivery of lipophilic, low-molecular-weight stable peptide (e.g., GLP-1 agonist analogues) and macrocycles with prolonged half-lives have been extensively explored.

## 11. Future Perspectives and Directions

The drug delivery of macromolecules through buccal mucosa is comparatively less investigated than other routes of administration. Solvent casting method is the most frequently used method for dissolving/dispersing actives in biocompatible polymeric films. However, there is a growing interest in 3D printing techniques using HME, fused deposition modelling and inkjet method. Even though the stability and permeability of macromolecules apparently increased compared to oral formulations the main drawback associated with film dosage form is difficulty of achieving high payload within the limited surface area of these mucoadhesive systems. Encapsulating with high payload in nanoparticles and embedding them in mucoadhesive polymeric may resolve this issue to certain extent. Investigations are currently progressing in the field of nanoparticles enabled buccal film and various functionalization strategies to allow permeation through the buccal mucosa and systemic targeting.

Finding new functional excipients such as thiolated polymers with potential for permeation enhancement, exploring new pathways for buccal permeation such as ion-pair strategy, increasing the drug loading, 3D printing methods to incorporate multiple drug combinations and compartmentalization to separate incompatible drugs are other novel areas of future research and developments in trans-buccal delivery systems. Successful design and development of microneedles patch to deliver 1 mg of human insulin and human growth hormone in the buccal cavity of swine in a short time (<30 s) has been demonstrated. Clinical trials in human volunteers indicated that microneedle patches applied on buccal surfaces could enhance patient compliance and promote the pain free delivery of biologics and other drugs particularly to pediatric, bed ridden and elderly populations [195]. The oral mucosa is an attractive site for vaccination, but a water rich environment can limit accurate dose delivery of vaccines. Ovalbumin dip coated on the tips of microneedle patch was found to deliver vaccine into the epithelium of the mice buccal mucosa in a short period of time compared to flat disk patch coated with ovalbumin substrate without microneedles [196]. The advent of nanohybrid materials as drug-delivery systems can hold the beneficial properties of their precursors and present additional advantages namely versatile methods for their production, improved mechanical and thermal stability, higher capacity of co-loading multiple drugs and diagnostic agents with diverse characteristics [193]. Hydrogel nanoparticles can significantly function as pharmaceutical carriers for buccal delivery by encapsulating oppositely charged low molecular- weight drugs and macromolecules such as oligo- and polynucleotides (siRNA, DNA) as well as proteins as targeting motifs.

## 12. Conclusions

Highly vascularized and immunologically competent buccal mucosa can be considered to be a feasible and attractive alternate delivery route for potent drugs with rapid onset of action, macromolecules and vaccines. However, the need for safe and effective permeation/absorption enhancers is essential for rapid advancement in the field of buccal drug delivery. New functionalization strategies to modify the surface of nanoparticles could transport different types of drugs efficiently through the buccal route.

## Figures and Tables

**Table 1:** List of penetration enhancers, transport mechanisms, and key findings.

Category	Examples	Transport Mechanism	Key Findings	References
Surfactants	Anionic: Sodium lauryl sulfate, sodium dodecanoate Cationic: Cetylpyridinium chloride	Disruption of intercellular lipids and integrity of protein Increase water solubility of drugs	Mucosal lipids might be extracted above critical micelle concentration therefore reducing the barrier properties of buccal mucosa	[58,59,60,61]
	Non-ionic: Polyoxyethylene-9-lauryl ether, nonylphenoxy poly oxyethylene, polysorbates (Tweens), sorbitan fatty acid esters (Spans), macrogol ethers (Brijs), macrogol esters (Myrjs)	Hydrophobic interaction between surfactant and keratin fibrils causes swelling of epithelium		
	Bile salts: Sodium taurocholate, sodium cholate, sodium deoxycholate, sodium taurodihydrofusidate, sodium taurodeoxycholate	Penetration into intercellular regions, increase fluidity, solubilization and extraction of lipids Interaction with keratin leads to disruption of corneocytes		
Fatty acids and their esters	Capric acid, caprylic acid, lauric acid, linoleic acid, linolenic acid, oleic acid, 2-octyldodecyl myristate, 1-[(*N*,*N*-dimethylamino)propan-2-yl]dodecanoate)	Interact with phospholipid domain and increase the membrane fluidity	A parabolic correlation observed between fatty acid lipophilicity and permeation enhancement Ability to diffuse through mucosa and interact with the lipid region is determined by fatty acid chain length Improve paracellular bioabsorption through transient opening of tight junctions	[62,63]
Cyclodextrins	α,β,γ cyclodextrins, methylated cyclodextrins	Disruption of intercellular lipids and integrity of protein	Molecular inclusion complex resulting in solubilization, lipid extraction and increasing buccal absorption	[64]
Polymers	Cationic: Chitosan, trimethyl chitosan, poly-L-arginine, L-lysine	Ionic interaction with negatively charged carboxyl and sulfate groups on mucin	Enhancement effect may be due to increasing the retention of the drug at the mucosal surface, which decrease the clearance of the drug by salivary flow Cationic cell penetrating peptide permit its interaction with anionic motifs on the mucin by a receptor-independent mechanism thus overcoming cell membrane impermeability and cellular internalization of actives	[65]
Chelating agents	Ethylenediaminetetraacetic acid, polyacrylate, citric acid, salicylates	The chelators form complexes with Ca^2+^ ions	Probably widen the gap between the cells and consequently facilitate paracellular transport of particularly, hydrophilic drugs	[66]
Miscellaneous	Azone (1-dodecylazacycloheptan-2-one)	Disrupts the lipid bilayers and increases the fluidity and permeation in the lipid regions of the biological barrier	Efficacy strongly dependent on its concentration (1–5%) and is also influenced by the choice of vehicle from which it is applied Effective for both hydrophilic and lipophilic drugs in polar medium	[67]

**Table 2:** Examples of mucoadhesive buccal patches and their characteristics.

Type	Polymer Constituents	Drugs Used	Manufacturing Method	Highlights	References
Controlled release	Carbopol, hydroxypropyl methylcellulose (HPMC), poloxamer and compritol 888 ATO	Lidocaine	Solvent casting	Free lidocaine and/or microspheres loaded patch fabricated using HPMC/carbopol and poloxamer Lidocaine microspheres prepared from Compritol 888 ATO employing spray congealing technique Change in formulation composition demonstrated to change the drug release mechanisms and able to provide either rapid, delayed or prolonged local anesthetic activity	[83]
Sustained release	Sodium alginate, HPMC, sodium carboxymethyl cellulose (NaCMC) and carbopol	Atenolol	Solvent casting	Patch prepared from sodium alginate Ex vivo permeation studies across goat buccal mucosa revealed 70.17 ± 2.28% release over a period of 24 h with maximum permeation flux (30.83 ± 1.23 μg/cm^2^/h) and minimum lag time (0.95 ± 0.22 h) Polymers used could provide sustained release of atenolol across porcine buccal mucosa for 24 h	[84]
Modified release	Xanthan gum, polyvinyl alcohol (PVA) and HPMC E-15	Zolmitriptan	Solvent casting	Bilayer patch prepared from xanthan gum In vitro drug release studies showed rapid drug release; 43.15% within 15 min, followed by sustained release rate over 5 h Incorporation of 4% dimethyl sulfoxide demonstrated 3.29-fold drug permeation, transported 29.10% of drug after 5 h	[85]
Immediate release	HPMC, PVA, polyvinylpyrrolidone and ethyl cellulose	Carbamazepine	Solvent casting	Water impermeable polypropylene backing layer provided unidirectional drug release Due to high water uptake, PEG 400 containing batches showed maximum in vitro release and increased mucoadhesion Drug release was controlled by either diffusion or non-Fickian diffusion	[86]
Peptide delivery	Chitosan, choline and geranic acid	Insulin	Solvent casting	Viscous gel made of choline and geranic acid sandwiched between two layers of chitosan Significant increase (7-fold) in the cumulative insulin transport across the ex vivo porcine buccal tissue was demonstrated (~26% of loaded insulin) In vivo studies in rat buccal pouch lowered blood glucose levels up to 50% in a dose dependent manner Serum insulin plateaued after 3 h for the duration of the study	[87]

**Table 3:** Examples of mucoadhesive buccal films based on their therapeutic category.

Therapeutic Classification	Polymer/Plasticizer	Active Ingredient	Manufacturing Method	Comments	References
Antihypertensive	Chitosan, polyvinylpyrrolidone, PVA, gelatin/propylene glycol	Propranolol HCl	Solvent casting	Personalized bilayered buccal film useful for pediatric population	[95]
Antifungal	Dextran, maltodextrin, HPMC, HPC/PEG 400 and glycerol	Amphotericin B	Solvent casting	Mechanical strength of the film was contributed by Avicel 200 and Avicel CL611 Physically stable orodispersible film was effective in oropharyngeal candidiasis	[96]
Antiepileptic	HPMC	Diazepam	*	Soluble film formulation of diazepam (Libervant™) effective in acute seizure emergencies Dose can be adjusted by cutting the film of suitable size	[97]
Antiprotozoal/anti-inflammatory	HPMC, PVA, chitosan/glycerin	Ornidazole and dexamethasone sodium phosphate	Solvent casting	Double layered film demonstrated >95% drug release in 4 h Significant effect on mucosal repair and reduced ulcer inflammation	[98]
Anesthetic/analgesic and anti-inflammatory/ mucolytic	HPMC, NaCMC, Chitosan/propylene glycol and sorbitol	Lidocaine HCl, benzydamine HCl, N-acetyl-cysteine	Solvent casting	Biocompatible bilayered mucoadhesive film stimulates cell proliferation and demonstrated therapeutic effect in buccal mucositis	[99]
Anti-inflammatory	HPMC, ethyl cellulose, chitosan, NaCMC, carbopol 971P/propylene glycol, PEG 8000	Fluticasone propionate	Solvent casting	Optimized formulation exhibited sustained drug release for 10 h Enhanced pharmacokinetic parameters was demonstrated compared to equivalent dose of mouthwash	[100]

* Undisclosed.

**Table 4:** Frequently used polymeric and lipid nanoparticles for buccal delivery and their characteristics.

Types of Nanoparticles	Nanoparticle Composition	Method	Polymers/Drug	Outcome	Key Points	References
Nanospheres	Poly (lactic-co-glycolic acid)	Double-emulsion solvent evaporation	HPMC K15 and Eudragit RS 100/selegiline	Potential to prolong retention, provide controlled release, enhance bioavailability	Buccal film fabricated from HPMC and eudragit embedded with poly (lac-tic-co-glycolic acid) nanospheres Permeation rate of selegiline mainly influenced by the film composition used The overall mean value of AUC0-α (2935.65 ± 194.24 ng.h/mL) from buccal film was found to be ~3 fold higher (*p* < 0.0001) as compared to oral solution	[124]
Nanoparticles	Poly (lactic-co-glycolic acid)	Double-emulsion solvent evaporation	Chitosan/ *C*-glycosyl flavonoid fraction of *Cecropia glaziovii*	Capacity to overcome low bioavailability of flavonoid extract	Dynamic mechanical analysis tests indicated that increasing of nanoparticles concentration caused decreased stiffness and an increased glass transition temperature Cytotoxic assay results indicated that these systems showed no cytotoxicity	[125]
Solid-lipid nanoparticles	Lipoid S100 and polysorbate 80	Solvent injection	HPMC/coumarin 6	Could be used for poorly aqueous soluble drugs	Lipid nanoparticles improved the cellular permeability through mucosal epithelial cells The quality of the solid-lipid nanoparticles loaded film and placebo mucoadhesive film were same	[126]
Liposomes	Polyvinylpyrrolidone	Electron spinning	Na CMC and chitosan/carvedilol	Initial burst release avoided with positive effect on permeation	Coaxial fibers-based self-assembling liposomes formed Demonstrated significant permeation across porcine TR146 cell culture and porcine buccal mucosa Cytotoxicity assay indicated absence of any toxicity caused by the fibers	[127]
Nanolipid structures	D-α-tocopherol PEG 1000 succinate, almond oil, compritol, phosphatidylcholine, gelucire 44/14	Hot emulsification–ultrasonication technique	Carbopol 934 and HPMC/glimepiride	Suitability to transport across buccal mucosa in sustained release manner	Selected concentration of micelles to nanostructured lipid carriers, carbopol and sodium cholate were 100%, 0.05% and 1.8%, respectively using a Box-Behnken design Optimized mucoadhesive film with a backing layer of ethyl cellulose demonstrated unidirectional glimepiride release of 93.9% at 6 h	[128]

**Table 5:** In vitro evaluation techniques typically employed for buccal film.

Technique	Principle	Evaluation Parameters	Ranges Units	References
Tensile test	The resistance of the thin strip of film against a dragging force is determined using a texture analyzer or modified balance method. Young modulus measures the deformation tendency of the film	Tensile strength = breaking force (N)/cross-sectional area (cm^2^) of the film The slope value from stress strain curve measures the Young modulus Percentage at the break, strain energy, energy to break can be calculated	16.6–24.3 MPa	[169,172]
Puncture test	The resistance of the thin film against the compression force until it breaks, cracks, or a desired loss in the force resisting the probe movement occurs	Toughness	0.2–13 mJ	[173,174]
Indentation test	Measure load as a function of penetration depth	Hardness and elastic modulus	1 mPa and ~100 mPa	[175]
Folding endurance	Repeatedly fold the film at 180° angle of the plane at the same plane until it breaks or folded to 300 times without breaking. The number of times the film is folded without breaking is computed as the folding endurance value	Flexibility	~300 count	[176]
Water absorption capacity	Swelling capacity assess bioadhesion behavior and drug release from the film	Percentage hydration is calculated by the equation [(W2 − W1) × 100/W1], where W1 weight of the film, W2 weight of the film after swelling in simulated saliva after predetermined time	5–25%	[177]
Thickness and weight variation	Thickness is determined using electronic digital micrometer, screw gauge, vernier caliper or by scanning electron microscopy images. Weight variation is calculated by subtracting weight of individual film from average weight and then divided by average weight of the film	Uniformity of the dose in the film	50–1000 μm and <50 mg	[169]
Surface morphology	Fixing the films on stubs, sputter coated with gold in an inert environment and imaged	Surface texture, pores, crystallinity, uniformity of drug distribution, thickness	-	[178]
Surface pH	Allowing it to swell by contact with distilled water for a short time (<2 h) at room temperature (25 °C)	pH at the area of application	6.0–7.5	[179]
Crystallinity	Place the sample in the sample holder of X-ray diffractometer and scan	Presence of crystalline or amorphous form of the sample	%	[180]
Thermal analysis	Heating the sample in aluminum pan at elevated temperature at uniform heating rate	Identify the existence of phase transition, recrystallization or molecular interaction of drug within the film	°C	[181]
Fourier-transform infrared spectroscopy	Specific ratio of drug and potassium bromide compressed at particular pressure and scanned	Drug-polymer interaction	cm^−1^	[182]
Mucoadhesive strength	Buccal film is attached to the probe of the texture analyzer using cyanoacrylate adhesive. Buccal epithelium of rabbit is fixed on the stationary platform of a texture analyzer. The probe of the texture analyzer was brought down gradually till the probe touch the mucosa	Adhesion strength is evaluated using shear stress, peel strength and tensile strength depending on the direction in which the mucoadhesive material is detached from the biological surface	6–7 N	[183]
In vitro drug release	Paddle over disc method using USPXXIV Type 2 apparatus	Release of drug from the prepared film using simulated saliva (pH 6.2)	%	[184]
Ex vivo permeation	Freshly excised buccal mucosa of rabbit using Franz diffusion cell, continuous flow diffusion cell, Ussing chamber, human buccal cell line (TR146), cell culture model	Establishing the absorption of drug across buccal epithelium by means of flux (J) and permeability coefficient (P)	J = μg/cm^2^/h P = cm/h	[17,181,185]

**Table 6:** Ongoing and completed clinical trials of buccal film formulations designed for systemic delivery.

Clinical Trials	Indication	Phase	Enrolment	Identifier
Buccal prochlorperazine (6 mg) plus 2 cc normal saline versus intravenous prochlorperazine (10 mg) 2 cc plus two saccharin absorbable placebo tablets	Migraine disorders	Phase III	80	NCT02779959
Diazepam buccal film (10 mg–17.5 mg based on body weight) administered on inner aspect of the following a low or high fatty meal versus diastat rectal gel (10 mg–20 mg based on body weight) following a moderate fatty meal	Epilepsy	Phase I and Phase II	31	NCT03953820
Palonosetron hydrochloride buccal film (0.25 mg and 0.5 mg) versus palonosetron hydrochloride, 0.25 mg/5 mL intravenous solution	Nausea with vomiting chemotherapy-induced	Phase II	22	NCT04592198
Montelukast buccal film, administered 10 mg once or 30 mg twice daily versus placebo buccal film administered once or twice daily	Alzheimer’s disease	Phase II	70	NCT03402503
A comparison of sublingual and buccal misoprostol regimens after mifepristone for mid-trimester abortion	Legally induced abortion	Phase IV	320	NCT02708446
Pharmacokinetic and pharmacodynamic study of three different doses (0.5 µg/kg, 0.75 µg/kg, and 1 µg/kg) of oral transmucosal dexmedetomidine	Sedation	Phase II and Phase III	36	NCT03120247
Single dose crossover study to compare the respiratory drive after administration of belbuca (300 μg, 600 μg and 900 μg), oxycodone (30 mg and 60 mg) and placebo	Respiratory depression	Phase 1	19	NCT03996694
Safety and efficacy study of NH004 films (intra oral) with tropicamide at different dose (0.3 mg,1 mg and 3 mg) for relief of sialorrhea symptoms in Parkinson’s disease patients versus placebo	Sialorrhea in Parkinson’s disease	Phase II	19	NCT00761137
A double-blind, placebo-controlled evaluation of the efficacy, safety and tolerability of BEMA™ fentanyl (bioerodible mucoadhesive soluble fentanyl citrate film) in the treatment of breakthrough pain in cancer subjects	Breakthrough pain in cancer	Phase III	152	NCT00293033
Long-term open-label safety study to evaluate EN3409 (BEMA^®^ Buprenorphine buccal film) at doses 300–900 μg	Low back pain, osteoarthritis, neuropathic pain	Phase III	303	NCT01755546

## Data Availability

The data presented in this study is contained within this article.

## References

[1] Gilhotra R.M., Ikram M., Srivastava S., Gilhotra N. (2014). A clinical perspective on mucoadhesive buccal drug delivery systems. J. Biomed. Res..

[2] Macedo A.S., Castro P.M., Roque L., Thomé N.G., Reis C.P., Pintado M.E., Fonte P. (2020). Novel and revisited approaches in nanoparticle systems for buccal drug delivery. J. Control Release.

[3] Birudaraj R., Mahalingam R., Li X., Jasti B.R. (2005). Advances in buccal drug delivery. Crit. Rev. Ther. Drug Carr. Syst..

[4] Sankar V., Hearnden V., Hull K., Juras D.V., Greenberg M.S., Kerr A.R., Lockhart P.B., Patton L.L., Porter S., Thornhill M. (2011). Local drug delivery for oral mucosal diseases: Challenges and opportunities. Oral Dis..

[5] Senel S., Hincal A.A. (2001). Drug permeation enhancement via buccal route: Possibilities and limitations. J. Control Release.

[6] Campisi G., Paderni C., Saccone R., Di Fede O., Wolff A., Giannola L.I. (2010). Human buccal mucosa as an innovative site of drug delivery. Curr. Pharm. Des..

[7] Sandri G., Ruggeri M., Rossi S., Bonferoni M.C., Vigani B., Ferrari F., Martins J.P., Santos H.A. (2020). Chapter 8—(Trans)buccal drug delivery. Nanotechnology for Oral Drug Delivery.

[8] Tran P.H.L., Duan W., Tran T.T.D. (2019). Recent developments of nanoparticle-delivered dosage forms for buccal delivery. Int. J. Pharm..

[9] Squier C., Brogden K. (2010). Human Oral Mucosa: Development, Structure and Function.

[10] Chen J., Engelen L. (2012). Food Oral Processing: Fundamentals of Eating and Sensory Perception.

[11] Berkovitz B.K., Moxham B.J., Linden R.W., Sloan A.J. (2010). Master Dentistry Volume 3 Oral Biology E-Book: Oral Anatomy, Histology, Physiology and Biochemistry.

[12] Nelson S.J. (2014). Wheeler’s Dental Anatomy, Physiology and Occlusion-E-Book.

[13] Shojaei A.H. (1998). Buccal mucosa as a route for systemic drug delivery: A review. J. Pharm. Pharm. Sci..

[14] Wertz P.W., Squier C.A. (1991). Cellular and molecular basis of barrier function in oral epithelium. Crit. Rev. Ther. Drug Carr. Syst..

[15] Wertz P.W. (2021). Roles of lipids in the permeability barriers of skin and oral mucosa. Int. J. Mol. Sci..

[16] Harris D., Robinson J.R. (1992). Drug delivery via the mucous membranes of the oral cavity. J. Pharm. Sci..

[17] Bierbaumer L., Schwarze U.Y., Gruber R., Neuhaus W. (2018). Cell culture models of oral mucosal barriers: A review with a focus on applications, culture conditions and barrier properties. Tissue Barriers.

[18] Groeger S., Meyle J. (2019). Oral mucosal epithelial cells. Front. Immunol..

[19] Frenkel E.S., Ribbeck K. (2015). Salivary mucins in host defense and disease prevention. J. Oral Microbiol..

[20] Sawada A., Wakabayashi N., Ona M., Suzuki T. (2011). Viscoelasticity of human oral mucosa: Implications for masticatory biomechanics. J. Dent. Res..

[21] Patel V.F., Liu F., Brown M.B. (2011). Advances in oral transmucosal drug delivery. J. Control Release.

[22] Laffleur F., Bernkop-Schnürch A. (2013). Strategies for improving mucosal drug delivery. Nanomedicine.

[23] Li L.D., Crouzier T., Sarkar A., Dunphy L., Han J., Ribbeck K. (2013). Spatial configuration and composition of charge modulates transport into a mucin hydrogel barrier. Biophys. J..

[24] Aframian D.J., Davidowitz T., Benoliel R. (2006). The distribution of oral mucosal pH values in healthy saliva secretors. Oral Dis..

[25] Nair A.B., Shah J., Jacob S., Al-Dhubiab B.E., Patel V., Sreeharsha N., Shinu P. (2021). Development of mucoadhesive buccal film for rizatriptan: In vitro and in vivo evaluation. Pharmaceutics.

[26] Madhav N.V., Shakya A.K., Shakya P., Singh K. (2009). Orotransmucosal drug delivery systems: A review. J. Control Release.

[27] Smart J.D. (2005). Buccal drug delivery. Expert Opin. Drug Deliv..

[28] Alqahtani M.S., Kazi M., Alsenaidy M.A., Ahmad M.Z. (2021). Advances in oral drug delivery. Front. Pharmacol..

[29] Giovino C., Ayensu I., Tetteh J., Boateng J.S. (2012). Development and characterisation of chitosan films impregnated with insulin loaded PEG-b-PLA nanoparticles (NPs): A potential approach for buccal delivery of macromolecules. Int. J. Pharm..

[30] Davies A., Mundin G., Vriens J., Webber K., Buchanan A., Waghorn M. (2016). The influence of low salivary flow rates on the absorption of a sublingual fentanyl citrate formulation for breakthrough cancer pain. J. Pain Symptom Manag..

[31] Rossi S., Sandri G., Caramella C.M. (2005). Buccal drug delivery: A challenge already won?. Drug Discov. Today. Technol..

[32] Martins J.P., Santos H.A. (2020). Nanotechnology for Oral Drug Delivery: From Concept to Applications.

[33] Hombach J., Bernkop-Schnürch A. (2010). Mucoadhesive drug delivery systems. Handb. Exp. Pharmacol..

[34] Chang R.-J., Gent A.N. (1981). Effect of interfacial bonding on the strength of adhesion of elastomers. I. Self-adhesion. J. Polym. Sci. Polym. Phys. Ed..

[35] Yang C., Xing X., Li Z., Zhang S. (2020). A Comprehensive review on water diffusion in polymers focusing on the polymer-metal interface combination. Polymers.

[36] Karoyo A.H., Wilson L.D. (2021). A review on the design and hydration properties of natural polymer-based hydrogels. Materials.

[37] Shinkar D.M., Dhake A.S., Setty C.M. (2012). Drug delivery from the oral cavity: A focus on mucoadhesive buccal drug delivery systems. PDA J. Pharm. Sci. Technol..

[38] Andrews G.P., Laverty T.P., Jones D.S. (2009). Mucoadhesive polymeric platforms for controlled drug delivery. Eur. J. Pharm. Biopharm..

[39] Anderson W.H., Coakley R.D., Button B., Henderson A.G., Zeman K.L., Alexis N.E., Peden D.B., Lazarowski E.R., Davis C.W., Bailey S. (2015). The relationship of mucus concentration (hydration) to mucus osmotic pressure and transport in chronic bronchitis. Am. J. Respir. Crit. Care Med..

[40] Jabbari E., Wisniewski N., Peppas N.A. (1993). Evidence of mucoadhesion by chain interpenetration at a poly (acrylic acid)/mucin interface using ATR-FTIR spectroscopy. J. Control Release.

[41] Laffleur F. (2014). Mucoadhesive polymers for buccal drug delivery. Drug Dev. Ind. Pharm..

[42] Bagan J., Paderni C., Termine N., Campisi G., Lo Russo L., Compilato D., Di Fede O. (2012). Mucoadhesive polymers for oral transmucosal drug delivery: A review. Curr. Pharm. Des..

[43] Ways T.M.M., Lau W.M., Khutoryanskiy V.V. (2018). Chitosan and Its derivatives for application in mucoadhesive drug delivery systems. Polymers.

[44] Jovanović M., Tomić N., Cvijić S., Stojanović D., Ibrić S., Uskoković P. (2021). Mucoadhesive gelatin buccal films with propranolol hydrochloride: Evaluation of mechanical, mucoadhesive, and biopharmaceutical properties. Pharmaceutics.

[45] Chaves P.D.S., Frank L.A., Frank A.G., Pohlmann A.R., Guterres S.S., Beck R.C.R. (2018). Mucoadhesive properties of Eudragit®RS100, Eudragit®S100, and poly(ε-caprolactone) nanocapsules: Influence of the vehicle and the mucosal surface. AAPS PharmSciTech.

[46] Collado-González M., González Espinosa Y., Goycoolea F.M. (2019). Interaction between chitosan and mucin: Fundamentals and applications. Biomimetics.

[47] Puri V., Sharma A., Kumar P., Singh I. (2020). Thiolation of biopolymers for developing drug delivery systems with enhanced mechanical and mucoadhesive properties: A review. Polymers.

[48] Schmitz T., Grabovac V., Palmberger T.F., Hoffer M.H., Bernkop-Schnürch A. (2008). Synthesis and characterization of a chitosan-N-acetyl cysteine conjugate. Int. J. Pharm..

[49] Catron N.D., Lee H., Messersmith P.B. (2006). Enhancement of poly(ethylene glycol) mucoadsorption by biomimetic end group functionalization. Biointerphases.

[50] Xu J., Soliman G.M., Barralet J., Cerruti M. (2012). Mollusk glue inspired mucoadhesives for biomedical applications. Langmuir ACS J. Surf. Colloids.

[51] Kim K., Kim K., Ryu J.H., Lee H. (2015). Chitosan-catechol: A polymer with long-lasting mucoadhesive properties. Biomaterials.

[52] Hu S., Pei X., Duan L., Zhu Z., Liu Y., Chen J., Chen T., Ji P., Wan Q., Wang J. (2021). A mussel-inspired film for adhesion to wet buccal tissue and efficient buccal drug delivery. Nat. Commun..

[53] Williams A.C., Barry B.W. (2012). Penetration enhancers. Adv. Drug Deliv. Rev..

[54] Morales J.O., Huang S., Williams R.O., McConville J.T. (2014). Films loaded with insulin-coated nanoparticles (ICNP) as potential platforms for peptide buccal delivery. Colloids Surf. B Biointerfaces.

[55] Aungst B.J. (2012). Absorption enhancers: Applications and advances. AAPS J..

[56] Iyer H., Khedkar A., Verma M. (2010). Oral insulin—A review of current status. Diabetes Obes. Metab..

[57] Ramadon D., McCrudden M.T.C., Courtenay A.J., Donnelly R.F. (2021). Enhancement strategies for transdermal drug delivery systems: Current trends and applications. Drug Deliv. Transl. Res..

[58] Park K., Kwon I.C., Park K. (2011). Oral protein delivery: Current status and future prospect. React. Funct. Polym..

[59] Nicolazzo J.A., Reed B.L., Finnin B.C. (2005). Buccal penetration enhancers—How do they really work?. J. Control Release.

[60] Som I., Bhatia K., Yasir M. (2012). Status of surfactants as penetration enhancers in transdermal drug delivery. J. Pharm. Bioallied Sci..

[61] Hassan N., Ahad A., Ali M., Ali J. (2010). Chemical permeation enhancers for transbuccal drug delivery. Expert Opin. Drug Deliv..

[62] Padula C., Pescina S., Nicoli S., Santi P. (2018). New insights on the mechanism of fatty acids as buccal permeation enhancers. Pharmaceutics.

[63] Jampilek J., Brychtova K. (2012). Azone analogues: Classification, design, and transdermal penetration principles. Med. Res. Rev..

[64] Palem C.R., Kumar Battu S., Gannu R., Yamsani V.V., Repka M.A., Yamsani M.R. (2012). Role of cyclodextrin complexation in felodipine-sustained release matrix tablets intended for oral transmucosal delivery: In vitro and ex vivo characterization. Pharm. Dev. Technol..

[65] Senel S., Kremer M.J., Kaş S., Wertz P.W., Hincal A.A., Squier C.A. (2000). Enhancing effect of chitosan on peptide drug delivery across buccal mucosa. Biomaterials.

[66] Sood A., Panchagnula R. (2001). Peroral route: An opportunity for protein and peptide drug delivery. Chem. Rev..

[67] Hou S.Y., Flynn G.L. (1997). Influences of 1-dodecylazacycloheptan-2-one on permeation of membranes by weak electrolytes. 1. Theoretical analysis of weak electrolyte diffusion through membranes and studies involving silicone rubber membranes. J. Pharm. Sci..

[68] Sonaje K., Chuang E.Y., Lin K.J., Yen T.C., Su F.Y., Tseng M.T., Sung H.W. (2012). Opening of epithelial tight junctions and enhancement of paracellular permeation by chitosan: Microscopic, ultrastructural, and computed-tomographic observations. Mol. Pharm..

[69] Zubareva A., Shagdarova B., Varlamov V., Kashirina E., Svirshchevskaya E. (2017). Penetration and toxicity of chitosan and its derivatives. Eur. Polym. J..

[70] Sandri G., Rossi S., Ferrari F., Bonferoni M.C., Muzzarelli C., Caramella C. (2004). Assessment of chitosan derivatives as buccal and vaginal penetration enhancers. Eur. J. Pharm. Sci..

[71] Caon T., Jin L., Simões C.M., Norton R.S., Nicolazzo J.A. (2015). Enhancing the buccal mucosal delivery of peptide and protein therapeutics. Pharm. Res..

[72] Renukuntla J., Vadlapudi A.D., Patel A., Boddu S.H., Mitra A.K. (2013). Approaches for enhancing oral bioavailability of peptides and proteins. Int. J. Pharm..

[73] Hao J., Heng P.W. (2003). Buccal delivery systems. Drug Dev. Ind. Pharm..

[74] Veuillez F., Kalia Y.N., Jacques Y., Deshusses J., Buri P. (2001). Factors and strategies for improving buccal absorption of peptides. Eur. J. Pharm. Biopharm..

[75] Netsomboon K., Suchaoin W., Laffleur F., Prüfert F., Bernkop-Schnürch A. (2017). Multifunctional adhesive polymers: Preactivated thiolated chitosan-EDTA conjugates. Eur. J. Pharm. Biopharm..

[76] Bernkop-Schnürch A., Krauland A.H., Leitner V.M., Palmberger T. (2004). Thiomers: Potential excipients for non-invasive peptide delivery systems. Eur. J. Pharm. Biopharm..

[77] Langoth N., Kalbe J., Bernkop-Schnürch A. (2003). Development of buccal drug delivery systems based on a thiolated polymer. Int. J. Pharm..

[78] Bernkop-Schnürch A., Guggi D., Pinter Y. (2004). Thiolated chitosans: Development and in vitro evaluation of a mucoadhesive, permeation enhancing oral drug delivery system. J. Control Release.

[79] Guggi D., Krauland A.H., Bernkop-Schnürch A. (2003). Systemic peptide delivery via the stomach: In vivo evaluation of an oral dosage form for salmon calcitonin. J. Control Release.

[80] Marschütz M.K., Caliceti P., Bernkop-Schnürch A. (2000). Design and in vivo evaluation of an oral delivery system for insulin. Pharm. Res..

[81] Targhotra M., Chauhan M.K. (2020). An overview on various approaches and recent patents on buccal drug delivery systems. Curr. Pharm. Des..

[82] Kelemen A., Katona B., Módra S., Aigner Z., Sebe I., Pintye-Hódi K., Zelkó R., Regdon G.J., Kristó K. (2020). Effects of sucrose palmitate on the physico-chemical and mucoadhesive properties of buccal films. Molecules.

[83] Cavallari C., Fini A., Ospitali F. (2013). Mucoadhesive multiparticulate patch for the intrabuccal controlled delivery of lidocaine. Eur. J. Pharm. Biopharm..

[84] Adhikari S.N., Nayak B.S., Nayak A.K., Mohanty B. (2010). Formulation and evaluation of buccal patches for delivery of atenolol. AAPS PharmSciTech.

[85] Shiledar R.R., Tagalpallewar A.A., Kokare C.R. (2014). Formulation and in vitro evaluation of xanthan gum-based bilayered mucoadhesive buccal patches of zolmitriptan. Carbohydr. Polym..

[86] Govindasamy P., Kesavan B.R., Narasimha J.K. (2013). Formulation of unidirectional release buccal patches of carbamazepine and study of permeation through porcine buccal mucosa. Asian Pac. J. Trop. Biomed..

[87] Vaidya A., Mitragotri S. (2020). Ionic liquid-mediated delivery of insulin to buccal mucosa. J. Control Release.

[88] Chun M.K., Cho C.S., Choi H.K. (2001). A novel mucoadhesive polymer prepared by template polymerization of acrylic acid in the presence of poloxamer. J. Appl. Polym. Sci..

[89] Escalona-Rayo C.F., Serrano-Castañeda P., López-Cervantes M., Escobar-Chávez J.J. (2020). Optimization of unidirectional mucoadhesive buccal patches based on chitosan and pluronic® F-127 for metoprolol controlled release: In vitro and ex vivo evaluations. J. Pharm. Innov..

[90] Saxena A., Tewari G., Saraf S.A. (2011). Formulation and evaluation of mucoadhesive buccal patch of acyclovir utilizing inclusion phenomenon. Braz. J. Pharm. Sci..

[91] Vishnu Y.V., Chandrasekhar K., Ramesh G., Rao Y.M. (2007). Development of mucoadhesive patches for buccal administration of carvedilol. Curr. Drug Deliv..

[92] Kraisit P., Limmatvapirat S., Nunthanid J., Sriamornsak P., Luangtana-Anan M. (2017). Preparation and characterization of hydroxypropyl methylcellulose/polycarbophil mucoadhesive blend films using a mixture design approach. Chem. Pharm. Bull..

[93] Montero-Padilla S., Velaga S., Morales J.O. (2017). Buccal dosage forms: General considerations for pediatric patients. AAPS PharmSciTech.

[94] Jacob S., Nair A.B., Patel V., Shah J. (2020). 3D printing technologies: Recent development and emerging applications in various drug delivery systems. AAPS PharmSciTech.

[95] Abruzzo A., Nicoletta F.P., Dalena F., Cerchiara T., Luppi B., Bigucci F. (2017). Bilayered buccal films as child-appropriate dosage form for systemic administration of propranolol. Int. J. Pharm..

[96] Serrano D.R., Fernandez-Garcia R., Mele M., Healy A.M., Lalatsa A. (2019). Designing fast-dissolving orodispersible films of amphotericin B for oropharyngeal candidiasis. Pharmaceutics.

[97] Rogawski M.A., Heller A.H. (2019). Diazepam buccal film for the treatment of acute seizures. Epilepsy Behav..

[98] Zhang C., Liu Y., Li W., Gao P., Xiang D., Ren X., Liu D. (2019). Mucoadhesive buccal film containing ornidazole and dexamethasone for oral ulcers: In vitro and in vivo studies. Pharm. Dev. Technol..

[99] Alves T.F.R., Rios A.C., da Silva Pontes K., Portella D.L., Aranha N., Severino P., Souto E.B., Gonsalves J.K.M., de Souza Nunes R., Chaud M.V. (2020). Bilayer mucoadhesive buccal film for mucosal ulcers treatment: Development, characterization, and single study case. Pharmaceutics.

[100] Ammar H.O., Ghorab M.M., Mahmoud A.A., Shahin H.I. (2017). Design and in vitro/in vivo evaluation of ultra-thin mucoadhesive buccal film containing fluticasone propionate. AAPS PharmSciTech.

[101] Bala R., Pawar P., Khanna S., Arora S. (2013). Orally dissolving strips: A new approach to oral drug delivery system. Int. J. Pharm. Investig..

[102] Montenegro-Nicolini M., Miranda V., Morales J.O. (2017). Inkjet printing of proteins: An experimental approach. The AAPS journal.

[103] Palem C.R., Dudhipala N.R., Battu S.K., Repka M.A., Rao Yamsani M. (2016). Development, optimization and in vivo characterization of domperidone-controlled release hot-melt-extruded films for buccal delivery. Drug Dev. Ind. Pharm..

[104] Vuddanda P.R., Alomari M., Dodoo C.C., Trenfield S.J., Velaga S., Basit A.W., Gaisford S. (2018). Personalisation of warfarin therapy using thermal ink-jet printing. Eur. J. Pharm. Sci..

[105] Musazzi U.M., Selmin F., Ortenzi M.A., Mohammed G.K., Franzé S., Minghetti P., Cilurzo F. (2018). Personalized orodispersible films by hot melt ram extrusion 3D printing. Int. J. Pharm..

[106] Ehtezazi T., Algellay M., Islam Y., Roberts M., Dempster N.M., Sarker S.D. (2018). The application of 3D printing in the formulation of multilayered fast dissolving oral films. J. Pharm. Sci..

[107] Salehi S., Boddohi S. (2019). Design and optimization of kollicoat ® IR based mucoadhesive buccal film for co-delivery of rizatriptan benzoate and propranolol hydrochloride. Mater. Sci. Engineering. C Mater. Biol. Appl..

[108] Repka M.A., Prodduturi S., Stodghill S.P. (2003). Production and characterization of hot-melt extruded films containing clotrimazole. Drug Dev. Ind. Pharm..

[109] Alhijjaj M., Bouman J., Wellner N., Belton P., Qi S. (2015). Creating drug solubilization compartments via phase separation in multicomponent buccal patches prepared by direct hot melt extrusion-injection molding. Mol. Pharm..

[110] Montenegro-Nicolini M., Morales J.O. (2017). Overview and future potential of buccal mucoadhesive films as drug delivery systems for biologics. AAPS PharmSciTech.

[111] Mortazavian E., Dorkoosh F.A., Rafiee-Tehrani M. (2014). Design, characterization and ex vivo evaluation of chitosan film integrating of insulin nanoparticles composed of thiolated chitosan derivative for buccal delivery of insulin. Drug Dev. Ind. Pharm..

[112] de Barros J.M.S., Scherer T., Charalampopoulos D., Khutoryanskiy V.V., Edwards A.D. (2014). A laminated polymer film formulation for enteric delivery of live vaccine and probiotic bacteria. J. Pharm. Sci..

[113] Borges O., Borchard G., Verhoef J.C., de Sousa A., Junginger H.E. (2005). Preparation of coated nanoparticles for a new mucosal vaccine delivery system. Int. J. Pharm..

[114] Uddin M.N., Allon A., Roni M.A., Kouzi S. (2019). Overview and future potential of fast dissolving buccal films as drug delivery system for vaccines. J. Pharm. Pharm. Sci..

[115] Kraan H., Vrieling H., Czerkinsky C., Jiskoot W., Kersten G., Amorij J.P. (2014). Buccal and sublingual vaccine delivery. J. Control Release.

[116] Song J.H., Nguyen H.H., Cuburu N., Horimoto T., Ko S.Y., Park S.H., Czerkinsky C., Kweon M.N. (2008). Sublingual vaccination with influenza virus protects mice against lethal viral infection. Proc. Natl. Acad. Sci. USA.

[117] Han J., Zhao D., Li D., Wang X., Jin Z., Zhao K. (2018). Polymer-based nanomaterials and applications for vaccines and drugs. Polymers.

[118] Mašek J., Lubasová D., Lukáč R., Turánek-Knotigová P., Kulich P., Plocková J., Mašková E., Procházka L., Koudelka Š., Sasithorn N. (2017). Multi-layered nanofibrous mucoadhesive films for buccal and sublingual administration of drug-delivery and vaccination nanoparticles—Important step towards effective mucosal vaccines. J. Control Release.

[119] Sun B., Xia T. (2016). Nanomaterial-based vaccine adjuvants. J. Mater. Chem. B.

[120] Hu Y., Hoerle R., Ehrich M., Zhang C. (2015). Engineering the lipid layer of lipid-PLGA hybrid nanoparticles for enhanced in vitro cellular uptake and improved stability. Acta Biomater..

[121] Hensley C., Zhou P., Schnur S., Mahsoub H.M., Liang Y., Wang M.X., Page C., Yuan L., Bronshtein V. (2021). Thermostable, dissolvable buccal film rotavirus vaccine is highly effective in neonatal gnotobiotic pig challenge model. Vaccines.

[122] Amorij J.P., Huckriede A., Wilschut J., Frijlink H.W., Hinrichs W.L. (2008). Development of stable influenza vaccine powder formulations: Challenges and possibilities. Pharm. Res..

[123] Kraisit P., Limmatvapirat S., Luangtana-Anan M., Sriamornsak P. (2018). Buccal administration of mucoadhesive blend films saturated with propranolol loaded nanoparticles. Asian J. Pharm. Sci..

[124] Al-Dhubiab B.E., Nair A.B., Kumria R., Attimarad M., Harsha S. (2016). Development and evaluation of buccal films impregnated with selegiline-loaded nanospheres. Drug Deliv..

[125] Santos T.C.D., Rescignano N., Boff L., Reginatto F.H., Simões C.M.O., de Campos A.M., Mijangos C.U. (2017). Manufacture and characterization of chitosan/PLGA nanoparticles nanocomposite buccal films. Carbohydr. Polym..

[126] Tzanova M.M., Hagesaether E., Tho I. (2021). Solid lipid nanoparticle-loaded mucoadhesive buccal films—Critical quality attributes and in vitro safety & efficacy. Int. J. Pharm..

[127] Chen J., Duan H., Pan H., Yang X., Pan W. (2019). Two types of core/shell fibers based on carboxymethyl chitosan and sodium carboxymethyl cellulose with self-assembled liposome for buccal delivery of carvedilol across TR146 cell culture and porcine buccal mucosa. Int. J. Biol. Macromol..

[128] Basahih T.S., Alamoudi A.A., El-Say K.M., Alhakamy N.A., Ahmed O.A.A. (2020). Improved transmucosal delivery of glimepiride via unidirectional release buccal film loaded with vitamin E TPGS-based nanocarrier. Dose Response Publ. Int. Hormesis Soc..

[129] Hanif M., Zaman M., Chaurasiya V. (2015). Polymers used in buccal film: A review. Des. Monomers. Polym..

[130] Leal J., Smyth H.D.C., Ghosh D. (2017). Physicochemical properties of mucus and their impact on transmucosal drug delivery. Int. J. Pharm..

[131] Hoogstraate A., Senel S., Cullander C., Verhoef J., Junginger H., Bodde H. (1996). Effects of bile salts on transport rates and routes of FITC-labelled compounds across porcine buccal epithelium in vitro. J. Control Release.

[132] Jacob S., Shirwaikar A., Srinivasan K., Alex J., Prabu S., Mahalaxmi R., Kumar R. (2006). Stability of proteins in aqueous solution and solid state. Indian J. Pharm. Sci..

[133] Al-Nemrawi N.K., Alsharif S.S., Alzoubi K.H., Alkhatib R.Q. (2019). Preparation and characterization of insulin chitosan-nanoparticles loaded in buccal films. Pharm. Dev. Technol..

[134] Jacob S., Nair A.B., Shah J., Sreeharsha N., Gupta S., Shinu P. (2021). Emerging role of hydrogels in drug delivery systems, tissue engineering and wound management. Pharmaceutics.

[135] Rai R., Alwani S., Badea I. (2019). Polymeric nanoparticles in gene therapy: New avenues of design and optimization for delivery applications. Polymers.

[136] Kim S.H., Mok H., Jeong J.H., Kim S.W., Park T.G. (2006). Comparative evaluation of target-specific GFP gene silencing efficiencies for antisense ODN, synthetic siRNA, and siRNA plasmid complexed with PEI-PEG-FOL conjugate. Bioconjugate Chem..

[137] Akbari V., Rezazadeh M., Minayian M., Amirian M., Moghadas A., Talebi A. (2018). Effect of freeze drying on stability, thermo-responsive characteristics, and in vivo wound healing of erythropoietin-loaded trimethyl chitosan/glycerophosphate hydrogel. Res. Pharm. Sci..

[138] Rezazadeh M., Jafari N., Akbari V., Amirian M., Tabbakhian M., Minaiyan M., Rostami M. (2018). A mucoadhesive thermosensitive hydrogel containing erythropoietin as a potential treatment in oral mucositis: In vitro and in vivo studies. Drug Deliv. Transl. Res..

[139] Fonseca-Santos B., Chorilli M. (2018). An overview of polymeric dosage forms in buccal drug delivery: State of art, design of formulations and their in vivo performance evaluation. Mater. Sci. Eng. C Mater. Biol. Appl..

[140] SreeHarsha N., Hiremath J.G., Sarudkar S., Attimarad M., Al-Dhubiab B., Nair A.B., Venugopala K.N., Asif A.H. (2019). Spray dried amorphous form of simvastatin: Preparation and evaluation of the buccal tablet. Indian J. Pharm. Educ. Res..

[141] Panda S. (2020). Formulation and evaluation by appling 32 (three squire) factorial design of lercanidipine hydrochloride buccal tablets with mucoadhesive polymers. Indian J. Pharm. Educ. Res..

[142] Zeng N., Seguin J., Destruel P.L., Dumortier G., Maury M., Dhotel H., Bessodes M., Scherman D., Mignet N., Boudy V. (2017). Cyanine derivative as a suitable marker for thermosensitive in situ gelling delivery systems: In vitro and in vivo validation of a sustained buccal drug delivery. Int. J. Pharm..

[143] Sharpe L.A., Daily A.M., Horava S.D., Peppas N.A. (2014). Therapeutic applications of hydrogels in oral drug delivery. Expert Opin. Drug Deliv..

[144] Hibbins A.R., Kumar P., Choonara Y.E., Kondiah P.P.D., Marimuthu T., Du Toit L.C., Pillay V. (2017). Design of a versatile pH-responsive hydrogel for potential oral delivery of gastric-sensitive bioactives. Polymers.

[145] Kamaly N., Yameen B., Wu J., Farokhzad O.C. (2016). Degradable controlled-release polymers and polymeric nanoparticles: Mechanisms of controlling drug release. Chem. Rev..

[146] Al-Dhubiab B.E., Nair A.B., Kumria R., Attimarad M., Harsha S. (2015). Formulation and evaluation of nano based drug delivery system for the buccal delivery of acyclovir. Colloids Surf. B Biointerfaces.

[147] Nair A.B., Shah J., Al-Dhubiab B.E., Jacob S., Patel S.S., Venugopala K.N., Morsy M.A., Gupta S., Attimarad M., Sreeharsha N. (2021). Clarithromycin solid lipid nanoparticles for topical ocular therapy: Optimization, evaluation and in vivo studies. Pharmaceutics.

[148] Naseri N., Valizadeh H., Zakeri-Milani P. (2015). Solid lipid nanoparticles and nanostructured lipid carriers: Structure, preparation and application. Adv. Pharm. Bull..

[149] Abd El Azim H., Nafee N., Ramadan A., Khalafallah N. (2015). Liposomal buccal mucoadhesive film for improved delivery and permeation of water-soluble vitamins. Int. J. Pharm..

[150] Chen J., Pan H., Yang Y., Xiong S., Duan H., Yang X., Pan W. (2018). Self-assembled liposome from multi-layered fibrous mucoadhesive membrane for buccal delivery of drugs having high first-pass metabolism. Int. J. Pharm..

[151] El-Samaligy M.S., Afifi N.N., Mahmoud E.A. (2006). Increasing bioavailability of silymarin using a buccal liposomal delivery system: Preparation and experimental design investigation. Int. J. Pharm..

[152] Bashyal S., Seo J.E., Keum T., Noh G., Choi Y.W., Lee S. (2018). Facilitated permeation of insulin across TR146 cells by cholic acid derivatives-modified elastic bilosomes. Int. J. Nanomed..

[153] Kotha R.R., Luthria D.L. (2019). Curcumin: Biological, pharmaceutical, nutraceutical, and analytical aspects. Molecules.

[154] Hazzah H.A., Farid R.M., Nasra M.M., Zakaria M., Gawish Y., El-Massik M.A., Abdallah O.Y. (2016). A new approach for treatment of precancerous lesions with curcumin solid-lipid nanoparticle-loaded gels: In vitro and clinical evaluation. Drug Deliv..

[155] Hazzah H.A., Farid R.M., Nasra M.M., El-Massik M.A., Abdallah O.Y. (2015). Lyophilized sponges loaded with curcumin solid lipid nanoparticles for buccal delivery: Development and characterization. Int. J. Pharm..

[156] Portero A., Teijeiro-Osorio D., Alonso M.J., Remuñán-López C. (2007). Development of chitosan sponges for buccal administration of insulin. Carbohydr. Polym..

[157] Kassem M.A., ElMeshad A.N., Fares A.R. (2015). Lyophilized sustained release mucoadhesive chitosan sponges for buccal buspirone hydrochloride delivery: Formulation and in vitro evaluation. AAPS PharmSciTech.

[158] Lv Q., Shen C., Li X., Shen B., Yu C., Xu P., Xu H., Han J., Yuan H. (2015). Mucoadhesive buccal films containing phospholipid-bile salts-mixed micelles as an effective carrier for cucurbitacin B delivery. Drug Deliv..

[159] Jones E., Ojewole E., Kalhapure R., Govender T. (2014). In vitro comparative evaluation of monolayered multipolymeric films embedded with didanosine-loaded solid lipid nanoparticles: A potential buccal drug delivery system for ARV therapy. Drug Dev. Ind. Pharm..

[160] Kraisit P., Hirun N., Mahadlek J., Limmatvapirat S. (2021). Fluconazole-loaded solid lipid nanoparticles (SLNs) as a potential carrier for buccal drug delivery of oral candidiasis treatment using the Box-Behnken design. J. Drug Deliv. Sci. Technol..

[161] Mura P., Maestrelli F., D’Ambrosio M., Luceri C., Cirri M. (2021). Evaluation and comparison of solid lipid nanoparticles (SLNs) and nanostructured lipid carriers (NLCs) as vectors to develop hydrochlorothiazide effective and safe pediatric oral liquid formulations. Pharmaceutics.

[162] Kraisit P., Sarisuta N. (2018). Development of triamcinolone acetonide-loaded nanostructured lipid carriers (NLCs) for buccal drug delivery using the box-behnken design. Molecules.

[163] Tetyczka C., Griesbacher M., Absenger-Novak M., Fröhlich E., Roblegg E. (2017). Development of nanostructured lipid carriers for intraoral delivery of Domperidone. Int. J. Pharm..

[164] Zhang H., Zhu Y., Qu L., Wu H., Kong H., Yang Z., Chen D., Mäkilä E., Salonen J., Santos H.A. (2018). Gold nanorods conjugated porous silicon nanoparticles encapsulated in calcium alginate nano hydrogels using microemulsion templates. Nano Lett..

[165] Rao S., Richter K., Nguyen T.H., Boyd B.J., Porter C.J., Tan A., Prestidge C.A. (2015). Pluronic-functionalized silica-lipid hybrid microparticles: Improving the oral delivery of poorly water-soluble weak bases. Mol. Pharm..

[166] Jacob S., Nair A.B., Shah J. (2020). Emerging role of nanosuspensions in drug delivery systems. Biomater. Res..

[167] Rana P., Murthy R.S. (2013). Formulation and evaluation of mucoadhesive buccal films impregnated with carvedilol nanosuspension: A potential approach for delivery of drugs having high first-pass metabolism. Drug Deliv..

[168] Pornpitchanarong C., Rojanarata T., Opanasopit P., Ngawhirunpat T., Patrojanasophon P. (2020). Clotrimazole nanosuspensions-loaded hyaluronic acid-catechol/polyvinyl alcohol mucoadhesive films for oral candidiasis treatment. J. Drug Deliv. Sci. Technol..

[169] Nair A.B., Kumria R., Harsha S., Attimarad M., Al-Dhubiab B.E., Alhaider I.A. (2013). In vitro techniques to evaluate buccal films. J. Control Release.

[170] Nair A., Morsy M.A., Jacob S. (2018). Dose translation between laboratory animals and human in preclinical and clinical phases of drug development. Drug Dev. Res..

[171] Berben P., Bauer-Brandl A., Brandl M., Faller B., Flaten G.E., Jacobsen A.C., Brouwers J., Augustijns P. (2018). Drug permeability profiling using cell-free permeation tools: Overview and applications. Eur. J. Pharm. Sci..

[172] Bodini R.B., Guimarães J.d.G.L., Monaco-Lourenço C.A., Aparecida de Carvalho R. (2019). Effect of starch and hydroxypropyl methylcellulose polymers on the properties of orally disintegrating films. J. Drug Deliv. Sci. Technol..

[173] Preis M., Knop K., Breitkreutz J. (2014). Mechanical strength test for orodispersible and buccal films. Int. J. Pharm..

[174] Walicová V., Gajdziok J., Pavloková S., Vetchý D. (2017). Design and evaluation of mucoadhesive oral films containing sodium hyaluronate using multivariate data analysis. Pharm. Dev. Technol..

[175] Eleftheriadis G.K., Ritzoulis C., Bouropoulos N., Tzetzis D., Andreadis D.A., Boetker J., Rantanen J., Fatouros D.G. (2019). Unidirectional drug release from 3D printed mucoadhesive buccal films using FDM technology: In vitro and ex vivo evaluation. Eur. J. Pharm. Biopharm..

[176] Kumria R., Nair A.B., Al-Dhubiab B.E. (2014). Loratidine buccal films for allergic rhinitis: Development and evaluation. Drug Dev. Ind. Pharm..

[177] Kumria R., Nair A.B., Goomber G., Gupta S. (2016). Buccal films of prednisolone with enhanced bioavailability. Drug Deliv..

[178] Al-Dhubiab B.E., Nair A.B., Kumria R., Attimarad M., Harsha S. (2019). Development and evaluation of nebivolol hydrochloride nanocrystals impregnated buccal film. Farmacia.

[179] Nair A.B., Al-ghannam A.A., Al-Dhubiab B.E., Hasan A.A. (2017). Mucoadhesive film embedded with acyclovir loaded biopolymeric nanoparticles: In vitro studies. J. Young Pharm..

[180] Maher E.M., Ali A.M., Salem H.F., Abdelrahman A.A. (2016). In vitro/in vivo evaluation of an optimized fast dissolving oral film containing olanzapine co-amorphous dispersion with selected carboxylic acids. Drug Deliv..

[181] Nair A.B., Al-Dhubiab B.E., Shah J., Jacob S., Saraiya V., Attimarad M., SreeHarsha N., Akrawi S.H., Shehata T.M. (2020). Mucoadhesive buccal film of almotriptan improved therapeutic delivery in rabbit model. Saudi Pharm. J. SPJ.

[182] Nair A.B., Al-Dhubiab B.E., Shah J., Vimal P., Attimarad M., Harsha S. (2018). Development and evaluation of palonosetron loaded mucoadhesive buccal films. J. Drug Deliv. Sci. Technol..

[183] Kumria R., Al-Dhubiab B.E., Shah J., Nair A.B. (2018). Formulation and evaluation of chitosan-based buccal bioadhesive films of Zolmitriptan. J. Pharm. Innov..

[184] Jug M., Hafner A., Lovrić J., Kregar M.L., Pepić I., Vanić Ž., Cetina-Čižmek B., Filipović-Grčić J. (2018). An overview of in vitro dissolution/release methods for novel mucosal drug delivery systems. J. Pharm. Biomed. Anal..

[185] Lin G.C., Leitgeb T., Vladetic A., Friedl H.P., Rhodes N., Rossi A., Roblegg E., Neuhaus W. (2020). Optimization of an oral mucosa in vitro model based on cell line TR146. Tissue Barriers.

[186] Karki S., Kim H., Na S.-J., Shin D., Jo K., Lee J. (2016). Thin films as an emerging platform for drug delivery. Asian J. Pharm. Sci..

[187] Bhagurkar A.M., Darji M., Lakhani P., Thipsay P., Bandari S., Repka M.A. (2019). Effects of formulation composition on the characteristics of mucoadhesive films prepared by hot-melt extrusion technology. J. Pharm. Pharmacol..

[188] Montenegro-Nicolini M., Reyes P.E., Jara M.O., Vuddanda P.R., Neira-Carrillo A., Butto N., Velaga S., Morales J.O. (2018). The effect of inkjet printing over polymeric films as potential buccal biologics delivery systems. AAPS PharmSciTech.

[189] Morales J.O., McConville J.T. (2011). Manufacture and characterization of mucoadhesive buccal films. Eur. J. Pharm. Biopharm..

[190] Irfan M., Rabel S., Bukhtar Q., Qadir M.I., Jabeen F., Khan A. (2016). Orally disintegrating films: A modern expansion in drug delivery system. Saudi Pharm. J. SPJ.

[191] Perumal V.A., Govender T., Lutchman D., Mackraj I. (2008). Investigating a new approach to film casting for enhanced drug content uniformity in polymeric films. Drug Dev. Ind. Pharm..

[192] Ortiz A.C., Morales J.O. (2020). Buccal delivery of nanoparticles. Mucosal Deliv. Drugs Biol. Nanoparticles.

[193] Barnhart S.D. (2008). Thin film oral dosage forms. Modified-Release Drug Delivery Technology.

[194] Morales J.O., Brayden D.J. (2017). Buccal delivery of small molecules and biologics: Of mucoadhesive polymers, films, and nanoparticles. Curr. Opin. Pharmacol..

[195] Caffarel-Salvador E., Kim S., Soares V., Tian R.Y., Stern S.R., Minahan D., Yona R., Lu X., Zakaria F.R., Collins J. (2021). A microneedle platform for buccal macromolecule delivery. Sci. Adv..

[196] Oh Y.J., Cha H.R., Hwang S.J., Kim D.S., Choi Y.J., Kim Y.S., Shin Y.R., Nguyen T.T., Choi S.O., Lee J.M. (2021). Ovalbumin and cholera toxin delivery to buccal mucus for immunization using microneedles and comparison of immunological response to transmucosal delivery. Drug Deliv. Transl. Res..

